# Development of a Feasible and Efficient In Vitro Rescue Protocol for Immature *Prunus* spp. Embryos

**DOI:** 10.3390/plants13212953

**Published:** 2024-10-22

**Authors:** Maria Casanovas, Elisabet Claveria, Ramon Dolcet-Sanjuan

**Affiliations:** 1 IRTA, Fruitcentre, Plant In Vitro Culture Laboratory, Fruticulture Program, Parc AgroBiotech, 25003 Lleida, Spain; 2 IRTA, Torre Marimon, Serveis Corporatius de Proximitat, Caldes de Montbui, 08140 Barcelona, Spain

**Keywords:** acclimatization, apricot, embryo rescue, flat fruits, nectarine, peach, vermiculite

## Abstract

The major factors affecting the in vitro immature embryo rescue efficiencies from *Prunus persica* or *P. armeniaca* accessions have been identified, along with improving the feasibility. Variations in the woody plant medium (WPM) were used depending on the embryo size. Embryos less than 5 mm long were cultured in WPM supplemented with 1 μM BAP and 1 μM GA_3_, while embryos bigger than 5 mm long were cultured in hormone-free medium, with or without vermiculite. The environmental in vitro culture conditions consisted of three phases: a (I) stratification at 4 °C during a 3- to 5-month-long period in the dark, followed by (II) growth of germinated embryos at 14 °C for a 4-week-long period, with 12 h light a day, which favors plantlet development, and finally, (III) growth at 24 °C, with 16 h light a day, until the plantlets were acclimatized in the greenhouse. The germination of smaller embryos, at the end of phase I, ranged from 82.2% to 22.1% for apricots and flat peaches, respectively, whereas for bigger embryos, the germination varied from 97.3% to 53.2% for the same species. The embryo germination for peaches and nectarines ranged from 40.1% to 30.3% for smaller embryos, and from 91.9% to 63.0% for bigger embryos. Endo- and epiphytic contamination, affecting from 7.4% to 52.9% of cultured embryos, depending on the fruit type and conservation conditions, and the capacity to acclimate to soil conditions, ranging from 50.4% to 93.2%, were the two most important factors influencing the protocol’s efficiency and feasibility. Considering the overall efficiencies, expressed as hardened plants transferred to field plots over clean uncontaminated embryo, the values ranged from 55.8% for nectarines, 54.0% for peaches, 45.6% for apricots, and 23.3% for flat fruits. The addition of vermiculite to the culture medium significantly improved the plantlet development, avoiding subculture to fresh medium when an extension of phase III was required before acclimatization. Compared to laboratory glassware, the use of food glass containers with air-permeable sealing film, along with vermiculite-containing medium, significantly reduced the costs when handling the large number of embryos required for breeding programs.

## 1. Introduction

Immature embryo rescue is one of the most widely used plant in vitro culture techniques for plant breeding. *Prunus* breeding programs use these protocols for the development of early ripening cultivars, starting from parents bearing fruits for which the flesh ripens faster than their embryos. These immature embryos fail to germinate in soil, following the mechanical treatments to remove the endocarp, cold stratification, and germination in pots in greenhouse conditions, which are useful in other *Prunus* varieties [1]. In vitro embryo rescue has been used since the early 1990s to improve the percentages of viable plant recovery from peach or nectarine [2,3,4,5,6,7,8,9,10,11], apricot [12], cherry [13] and interspecific hybrids [14,15,16], oriented to generate low chilling, early ripening, new varieties for the worldwide commercial fruit production, particularly in Spain [17].

The first works on peach in vitro embryo rescue were focused on in-ovule culture [18,19,20,21] to facilitate embryo development, surrounded by its seed coat and endosperm, prior to embryo excision, and cultured in media wholly composed of substances that are chemically known. Since higher embryo rescue efficiencies were obtained for later fruit maturity stages, and because peach and nectarine breeding programs need to manipulate large numbers of embryos, direct immature embryo culture was generally adopted in more recent works, even without removing the embryo integuments [22].

In most works describing in vitro embryo rescue protocols, seeds were dissected out of fruits, and after disinfection and removal of the seed coat and endosperm, embryos were cultured in glass tubes with semisolid agar-containing medium. Herein, an alternative medium containing vermiculite, previously used as a substrate to enhance the rooting of diverse recalcitrant species, such as apple rootstocks and walnuts [23,24,25], chestnuts [26], hybrid tea roses [27], and several ornamental *Prunus* spp. [28], and for peaches and nectarines embryo rescue [5,29], is extensively used for embryo rescue and in vitro maintenance of plantlets, without subculture to fresh medium, until the external climatic conditions are appropriate for acclimatization and hardening to greenhouse conditions, which is an important step prior to field plantation and selection.

Conventional container sealing films, such as Parafilm [30], or limited flask ventilation [31] have been demonstrated to induce stress reactions in some plants. Therefore, an appropriate flask and cap combination for proper ventilation represents an important factor for proper in vitro plant development, as shown in the rooting of apricot [32,33], sugarcane [34], and sweet chestnut [35]. Laboratory test tubes, along with caps and appropriate racks to hold them, are an important limiting expense when the number of embryos to rescue rises to several thousand a year. A more economic, alternative glass flask, used in the asparagus canning industry, along with an autoclavable oxygen-permeable sealing film to guarantee proper embryo germination and plantlet growth, are described herein.

The results reported so far [2,3,4,5,6,7,8,9,10,11,12,13] rarely mention the incidence of in vitro embryo contamination due to external or endophytic microorganisms, scaping seed disinfection. In the present work, the importance of this value and the need to reduce the incidence to improve the overall embryo rescue efficiency are reported.

Works published on *Prunus* spp. in vitro embryo rescue have been focused on measuring the embryo germination capacities of specific crosses. The objective of the present work was to determine the importance of additional factors in relation to the feasibility of an embryo rescue protocol. The fruit type effect on the germination capacity was hypothesized to influence the overall immature embryo rescue efficiencies, along with in vitro embryo contamination and the embryo sizes. The presence of vermiculite in the culture medium was hypothesized to be an influencing factor in the applicability of the whole process. Therein, the accumulated results of eight consecutive seasons are reported, providing results for the whole process, from embryo culture to in vitro plantlet development, and later to acclimatization and hardening under greenhouse conditions, for a broad range of fruit types, including peaches, nectarines, flat peaches, flat nectarines, and apricots.

## 2. Results

### 2.1. Contamination and Embryo Size Depending on the Fruit Type

**Figure 1:**
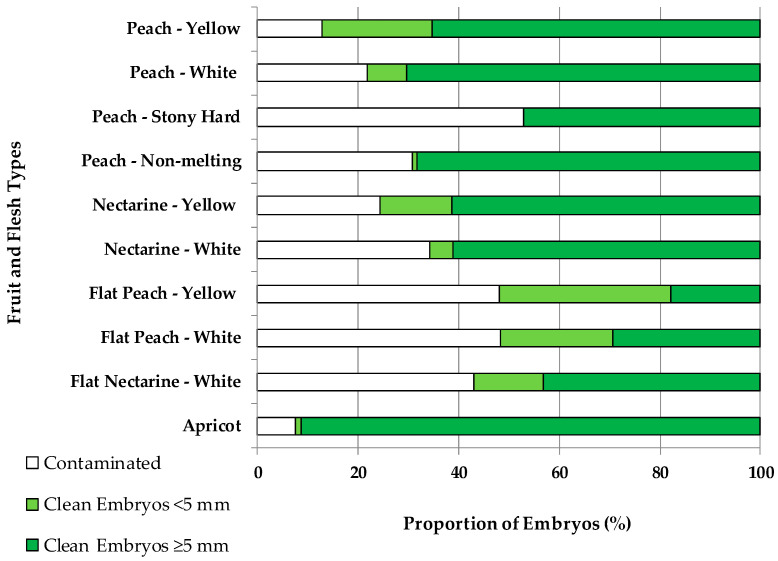
Percentage of contaminated embryos, clean embryos < 5 mm, and clean embryos ≥ 5 mm over the total number of embryos cultured in vitro for different fruit and flesh types.

In vitro contamination by exogenous and endogenous microorganisms was found to be dependent on the fruit type (Figure 1). Apricots (7%), peaches with yellow (13%) and white flesh (22%), nectarines with yellow flesh (24%), peaches with non-melting flesh (31%), and nectarines with white flesh (34%) had the lowest percentages of contamination of the cultured embryos. Instead, the highest percentages of contamination were found on flat nectarines (43%) and flat peaches, with either yellow or white flesh (48%), and on stony hard peaches (53%).

The embryo sizes were also dependent on the fruit type. The highest percentage (34%) of embryos < 5 mm was found in flat peaches with yellow flesh. Flat peaches with white flesh and peaches with yellow flesh both had 22% of embryos < 5 mm. Nectarines with yellow flesh and flat nectarines with white flesh both had 14% of embryos < 5 mm. The rest of the fruit types had lower percentages of small embryos, such as 8% in peaches with white flesh or 5% in nectarines with white flesh, only 1% for apricots and peaches with non-melting flesh, and none for stony hard peaches. Considering the 274 crosses, there was no significant correlation (R^2^ = 0.19) between the level of contamination and the percentage of embryos < 5 mm. In addition, there was a significantly (*p* = 0.01) lower level of contamination (23.4%) for small embryos than for those ≥ 5 mm (29.1%).

### 2.2. Embryo Germination Percentages Depending on the Fruit Type and Embryo Size

**Figure 2:**
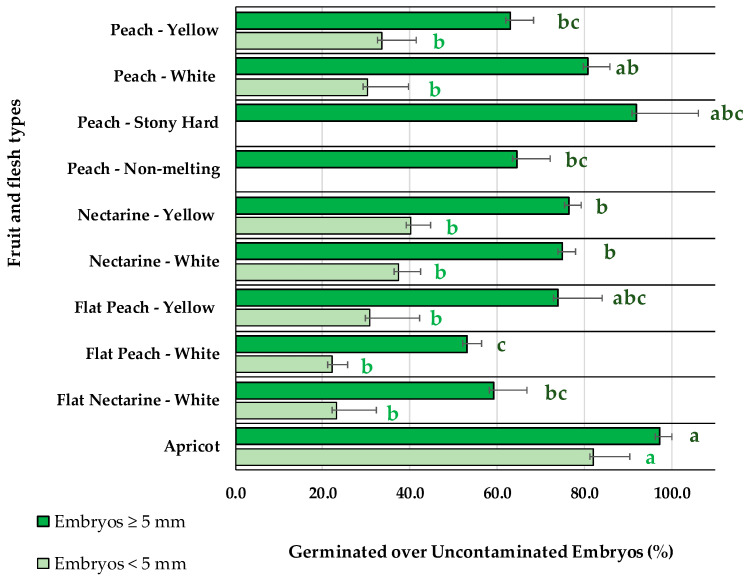
Percentage of germinated over uncontaminated embryos, of sizes <5 mm or ≥5 mm, for different fruit and flesh types. Bars represent the standard error. Means with the same letter within each embryo size are not significantly different according to Tukey’s HSD (*p* = 0.05).

After stratification at 4 °C, the embryo germination for different fruit types (Table 1), accounted as germinated embryos over uncontaminated embryos (Figure 2), was significantly lower (*p* < 0.001) for small embryos (<5 mm) than for big embryos (≥5 mm) in all the fruit types except for apricot. For small embryos (<5 mm), no statistically significant differences were observed among peaches and nectarines, with values ranging from 22.1%, for flat peaches with white flesh, to 40.1%, for nectarines with yellow flesh, while apricots had significantly higher embryo germination percentages (82.2%). Instead, for big embryos (≥5 mm), significant differences were observed among some fruit types. Higher embryo germination efficiencies were observed for apricots (97.3%). An intermediate level of germination was observed for nectarines with white (75.0%) or yellow flesh (76.5%). The lowest germination percentage was registered for flat peaches with white flesh (53.2%). The rest of the fruit types, such as flat nectarines with white flesh (59.3%), peaches with yellow flesh (63.0%), flat peaches with yellow flesh (74.0%), peaches with white flesh (80.7%), non-melting peaches (64.6%), and stony hard peaches (91.9%), were statistically not different due to the diverse number of crosses and fruits available from each fruit type (Table 1).

### 2.3. Embryo Germination Percentages Depending on the Use of Vermiculite

**Figure 3:**
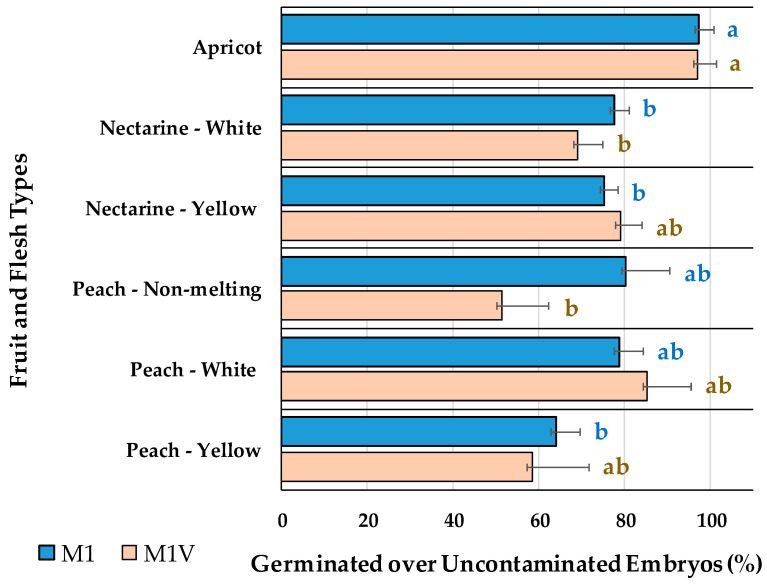
Percentage of germinated over uncontaminated embryos, for different fruit types, cultured in M1 or M1V media. Bars represent the standard error. Means with the same letter within each medium are not significantly different according to Tukey’s HSD (*p* = 0.05).

The embryo germination on M1, with no vermiculite, compared to M1V, with vermiculite, assayed for embryos bigger than 5-mm-long is presented in Figure 3. Flat fruits were not included in this comparison, since though some of their embryos were bigger than 5 mm, they all had a friable structure, with three cotyledons, easily broken and separated from the embryo axis. The addition of vermiculite to the medium had no statistically significant (*p* = 0.188) effect on the germination percentages of apricots, nectarines or peaches with either white or yellow flesh, or on non-melting peaches (Figure 3). Apricots had higher germination percentages than nectarines with white flesh, cultured in either of the two media. When cultured in M1 medium, peaches with white flesh and non-melting peaches had germination percentages comparable to apricots. Using vermiculite in the culture medium (M1V) increased the germination percentages of nectarines with yellow flesh, peaches with white flesh and peaches with yellow flesh to the values observed in apricots.

### 2.4. Effect of Vermiculite on In Vitro Plantlet Growth

**Figure 4:**
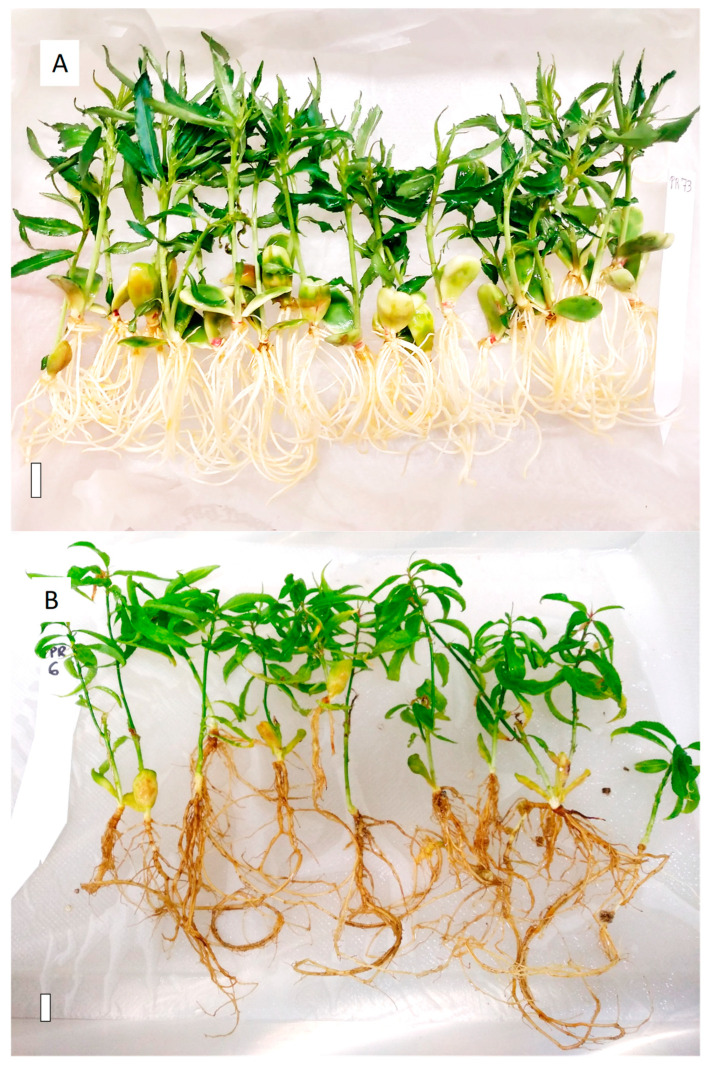
Plantlets from embryos grown in M1 (**A**) and in M1V medium (**B**). Solid bar is equivalent to 1 cm length.

Differential plantlet growth in the in vitro culture chamber was observed in M1 when compared with M1V, with vermiculite, as shown in Figure 4. Both media generated homogeneous plantlets, which could be acclimated to soil. However, the plantlets grown in M1V (Figure 4B) compared to M1 had longer shoots and roots, with much better developed leaves. The M1V-derived plants had more flexible root system, with thinner roots and abundant secondary roots, compared to the plants derived from M1 medium, whose roots were thicker and friable (Figure 4A). The root structure of plantlets grown in M1V were much more easily removed from the tubes, cleaned, manipulated, and transplanted to soil, without breakage, which facilitated acclimatization and hardening in the greenhouse. The aerial part of the M1V-derived plantlets was larger in volume, with broader expanded leaves, which were hardened in vitro, facilitating the acclimatization process to soil. In contrast, some of the plantlets grown in M1 medium presented vitrification, and consequently, their acclimatization was not viable.

### 2.5. Embryo Rescue and Viable Plant Production Efficiencies for Different Fruit Types

**Figure 5:**
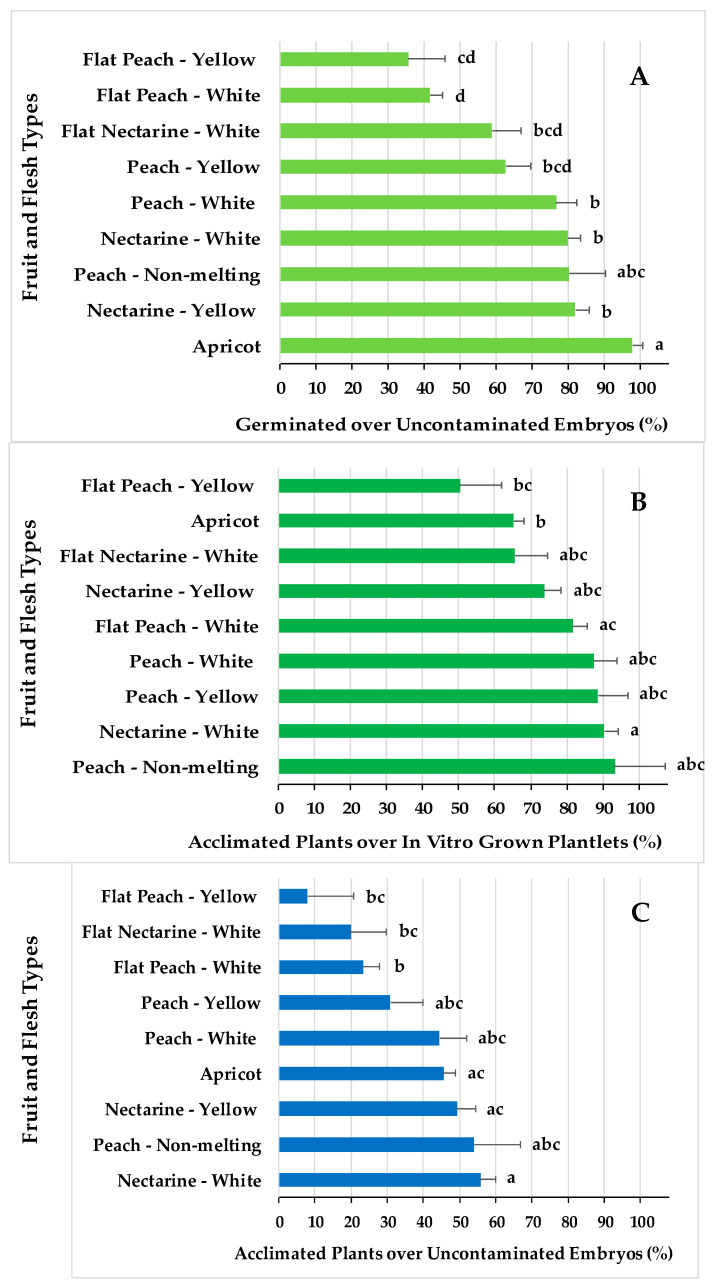
Embryo rescue efficiencies in percentages of (**A**) in vitro germinated over uncontaminated embryos, (**B**) acclimated plants in the greenhouse over the in vitro developed plantlets, and (**C**) acclimated plants over uncontaminated embryos. Means with the same letter within each efficiency rate are not significantly different according to Tukey’s HSD (*p* = 0.05).

The percentage of in vitro embryo germination over uncontaminated embryos (Figure 5A) and the percentage of acclimated plants over the in vitro grown plantlets (Figure 5B) were the two efficiency indexes for which significant differential responses were found among the fruit types. The overall efficiency of the whole process, expressed as the percentage of acclimated plants over uncontaminated embryos, is shown Figure 5C for the different fruit types. This index shows the production of viable *Prunus* spp. plants over uncontaminated embryos transferred to the breeding programs.

The highest efficiency in terms of embryo germination (Figure 5A) observed for apricots was associated with a low efficiency in acclimatization to soil (Figure 5B), which consequently implied an intermediate overall embryo rescue efficiency (Figure 5C). The overall lower efficiencies in producing embryo rescued plantlets (Figure 5C) for flat peaches and flat nectarines, with white or yellow flesh, is associated with both the lowest embryo germination rates (Figure 5A) and lower or intermediate acclimatization ratios (Figure 5B). Nectarines with white flesh are the fruit types with the highest viable plant production ratio (Figure 5C), which corresponds with the highest acclimatization rations (Figure 5B) and intermediate embryo germination efficiencies (Figure 5A). Peaches, with either white or yellow flesh, had intermediate plant production efficiencies (Figure 5C), associated with intermediate acclimatization ratios (Figure 5B) and intermediate embryo germination efficiencies (Figure 5A).

## 3. Discussion

### 3.1. Contamination and Embryo Size Depending on the Fruit Type

In vitro contamination during embryo rescue was not usually presented in previous works [6,13,22,36], although there is a need to improve the rescue efficiencies, reducing the loses caused by epiphytic and endophytic microorganisms.

Improving the fruit harvest and storage conditions until embryo rescue was found to be a requirement. The fruit and flesh types, their maturity stage at harvest time, and the conservation conditions until seed extraction determined their pericarp integrity and softness. Flat peaches and nectarines, nectarines with white flesh, and non-melting peaches have abundant split endocarps and some cracked mesocarps at embryo rescue time, a morphological disorder related to the poor adaptation of flat peaches and nectarines to climatic conditions different to the climate of their center of origin in Asia [17]. Consequently, the seeds were easily contaminated by microorganisms, and their surface sterilization could not avoid the in vitro contamination (Figure 1). When the exocarp and endocarp were over ripened, not even the endocarp integrity of stony hard peaches could guaranty a low in vitro contamination. The results indicated that when the fruits were harvested one or two weeks prior to their commercial harvest, the pericarp integrity facilitated the reduction of in vitro embryo contamination, as for apricots, peaches with yellow or white flesh, and nectarines with yellow flesh. Future efforts should focus on improving seed disinfection or using beneficial microorganisms [37,38,39] for the biological control of harmful epi- and endophytic microorganisms.

The embryo size at fruit harvest was determined by the fruit type, with a higher proportion of small embryos (<5 mm) for flat peaches, peaches with yellow flesh, flat nectarines with white flesh, and nectarines with yellow flesh. The rest of the fruit types had lower proportions of small embryos, which explained the overall higher embryo rescue efficiencies for the studied fruit types (Figure 5C).

### 3.2. Embryo Germination Percentages Depending on the Fruit Type and Embryo Size

In the present study, in which 274 crosses from 10 different *Prunus* fruit types have been tested (Table 1), lower in vitro germination percentages were observed for smaller embryos (<5 mm) (Figure 2), as was reported previously in peach cultivars [40]. Some works proposed that embryos should be rescued before abortion and at the same time postponed until the embryos were big enough, particularly in interspecific hybrids involving *Prunus persica*, *P. armeniaca*, *P. salicina*, and *P. cerasifera* [14] and *P. salicina* and *P. armeniaca* [16]. In accordance with this, some works [9] using the number of weeks after pollination as an indicator for peach embryo development, without specifying the embryo size, found the maximum germination percentages at the maximum days after pollination (75 and 85 days). In accordance with this, in the present work, fruits were harvested at the maximum number of days after pollination, right before commercial ripening, saving the pericarp integrity.

The in vitro germination rates of small embryos (<5 mm) were found to be 82.2% in apricots, 40.1% in nectarines, 30% in peaches, and 22.1% in flat fruits, slightly better than the 30% reported for hybrids involving *P. cerasifera* and *P. armeniaca* [41]. Such germination improvement for small embryos as found in the present work could be explained by the use of M2 medium, which was supplemented with GA_3_, known to break the dormancy and promote embryo germination, and BAP, known to promote cell division [2,42]. The hormonal combination of 1 μM GA_3_ + 1 μM BAP present in the M2 medium was determined in previous works in our laboratory with small embryos (<5 mm) to be significantly better in promoting the germination of nectarines and flat peaches compared to the WPM without hormones, or other hormonal combinations ((I) 1 μM GA_3_; (II) 1 μM BAP; (III) 1 μM BAP + 1 μM Kinetin; or (IV) 1 μM GA_3_ + 1 μM BAP + 1 μM Kinetin).

The low embryo germination rates in flat fruits and nectarines (Figure 2) might be associated with high abortion ratios due to an abnormal endosperm development [43] and carbohydrate metabolism [44]. On the contrary, the apricot embryo rescue efficiencies were much higher than other fruit types, reaching highest efficiencies in disinfection and germination. The high in vitro germination ratios were in accordance with other studies [12,21], for which removing the seed coat improved the germination [1]. Apricots encountered more difficulties than peaches, nectarines, and flat fruits during acclimatization to soil, associated with shoot tip necrosis as a response to lack of calcium in the medium [45] and the high sensitivity of apricot plants to root asphyxia and soil waterlogging [46,47].

Abscisic acid (ABA) accumulation in the seed during embryo maturation, a germination inhibitor [22], was proposed as the explanation for the poorer germination of big apricots [12] and *Prunus avium* [13] embryos compared to the small embryos. The present study shows, for any fruit type tested, better germination percentages with the big (>5 mm) than the small (<5 mm) embryos (Figure 2), presumably caused by the seed coat and endosperm removal before in vitro embryo culture, the stratification length at 4 °C, and the culture at 14 °C after stratification.

The launch in the present study of a 14 °C in vitro culture period for 4 weeks, right after stratification at 4 °C, instead of direct culture to 24 °C, improved the embryo rescue efficiency, which is in accordance with previous works [19,36] recommending lower culture temperatures (18 to 20 °C) for better plantlet development. At 14 °C, even though it is not optimal for in vitro *Prunus* spp. micropropagation, the axis started to elongate, formed roots and shoots, and leaves grew slower than at 24 °C, avoiding the apical shoot-tip necrosis observed when germinated embryos were moved directly from 4 °C to 24 °C. Apical necrosis has been related to multiple factors, such as low boron and calcium content in the culture medium, and physical conditions, such as humidity and temperature, that guarantee the translocation of nutrients from the base to the apex [45,48,49]. Periodic reduction of the relative humidity in the in vitro culture flask induced leaf transpiration, root calcium uptake and translocation to the shoot, while constant high relative humidity reduced calcium movement [50]. Culturing embryos at 14 °C allowed for slower growth, less calcium demand and alleviated the incidence of shoot-tip necrosis.

### 3.3. Embryo Germination Percentage Depending on the Use of Vermiculite

In this work, vermiculite has been evaluated as an embryo support for the in vitro culture of *Prunus* spp. in an only-one-medium used from the initial immature embryo culture to the acclimatization of the resulting plantlets. Vermiculite-containing medium had previously been reported as a suitable substrate for rooting during the greenhouse acclimatization of chestnut [26], tangerine [51], peach [52] and aloe [53], and for the in vitro rooting improvement of walnut [25], grape [54], hybrid tea rose [27] and peach [28].

Earlier, the in vitro embryo rescue of 350 [5] and 600 [29] nectarines and peaches, replacing agar with vermiculite, demonstrated a higher percentage of germination with vermiculite than other potting substrates (coconut fiber or charcoal rice husk). These works reported high germination percentages (80–100%) in vermiculite, which were better than in medium with agar. In opposition to what former works found, herein, no statistically significant differences were observed between the M1 and M1V media in relation to the in vitro embryo germination of apricots, nectarines, or peaches (Figure 3). Therefore, the use of vermiculite in the medium (M1V) for embryos bigger than 5 mm long did not influence the germination efficiency of the studied *Prunus* spp. but helped with laboratory logistics and reduced labor inputs. Flat peaches or nectarines were not cultured in M1V, only in agar-containing media (M1 or M2), due to their embryo size and friable morphology.

### 3.4. Effect of Vermiculite on In Vitro Plantlet Growth

The use of vermiculite in the culture medium (M1V) presented advantages during the in vitro plantlet development and transfer to soil conditions, in accordance with what was reported before [25,26,27,28]. When the plantlets grew in medium with agar as a gelling agent (M1), the roots twisted on themselves following the tube’s shape and sometimes the shoot became chlorotic or vitrified when kept in the same recipient for a long time. In contrast, plantlets derived from vermiculite-containing medium (M1V) had significantly better root and shoot development. In M1V medium, roots ramified and produced secondary roots, presumably caused by the physical obstacle that vermiculite represents and the abundance of aerated spaces with higher oxygen and water levels [23,55]. Consequently, the plantlets in M1V developed taller shoots with bigger broader leaves (Figure 4). A better rhizosphere and aerial part favored successful acclimatization compared to the plantlets grown in agar-containing medium [23,25]. Vermiculite-containing medium facilitated the application of rhizosphere microorganisms during embryo rescue, and although the rescue efficiencies were not significantly increased, plantlet development and acclimatization to soil were improved [56].

A beneficial effect of the use of vermiculite in the culture medium (M1V), which was not mentioned before in the literature [25,26,27,28,51,53], was that the plantlets could be maintained in the same vessel for up to 8 additional weeks without subculture to fresh medium but by adding liquid PRU0 medium to the flask. This supposed lower labor and materials costs. Prolonging the plants’ life in the culture room, the acclimatization was time flexible, resolving the existence of adverse climatic conditions or difficulties in hand labor logistics in the laboratory. The advantages of M1V over M1 medium go along with the use of cheaper vessels and closures, alternative to the laboratory glass tubes, caps, and racks. In agreement to our results, improving the aeration of the in vitro culture recipients has also been shown to increase the number of usable potatoes microtubes [57] and the regeneration and growth of pepper plantlets [58] and sugarcane [34], and it increased the number and leaf size during acclimatization in chestnut plantlets [35]. A good vessel ventilation reduced the stress reactions of *Arabidopsis* probably caused by the high ethylene and carbon dioxide concentrations in the Petri dishes closed with air-tight tapes [30,31]. *Prunus* GF677 had the best multiplication and elongation in round vessels with a filter breathing strip [59]. Different jar closures were compared for the rooting and acclimatization of apricot plantlets [32,33], and the high ethylene concentration in the vessel with hermetic closure affected the growth of the plant cultures and was associated with lower root numbers and reduced plant survival in the acclimatization phase. Previous assays with *Pyrus* and *Prunus* plantlets in coculture with microorganisms demonstrated a CO_2_ accumulation of over 2% in the culture vessels [60], which proved to be toxic for plantlet development. Consequently, the vessels and caps described herein, widely used in the agri-food industry, substantially reduced the cost compared to the laboratory test tubes and proved to be optimal for embryo rescue.

### 3.5. Embryo Rescue and Viable Plant Production Efficiencies for Different Fruit Types

As commonly found in any plant in vitro culture methodology, the genotype, herein indicated as the fruit type, has a significant differential effect on the in vitro embryo rescue from the germination to acclimatization (Figure 2 and Figure 5). A major part of the studies on embryo rescue have been focused on the different culture conditions to improve germination in peaches and nectarines, showing the differential responses of the varieties [4,8,9]. Herein, an optimized protocol, over several seasons in our conditions, has been applied to different fruit types. The germination ratios presented in this work in peach and nectarine are like those published previously [7,8,22].

The in vitro embryo germination values reported here were comparable to those cited before [1,2,3,4,5,6,7,8,9,10,11,12,13,14,15,16]; however, the differential aspect of the protocol presented herein is its applicability to *Prunus* spp. breeding programs with a great number of embryos to be handled. Few studies have reported results concerning the differential acclimatization of the plantlets resulting from the embryo rescue [56]. In the present work, the results came from a larger number of embryos (Table 1) than in previous works [52], derived from different fruit types, varieties, and seasons. Former works reported a high percentage of acclimatization in peach, but after using a rooting medium before acclimatization, which complicated its application to programs involving many embryos.

A novelty of the results presented herein is the demonstration that the differential in vitro contamination by epiphytic and endophytic microorganisms (Figure 1), the different capacities for in vitro embryo germination (Figure 5A), and the acclimatization capacities to soil (Figure 5B) explained the overall ratios for plant production for the different *Prunus* spp. fruit types (Figure 5C).

## 4. Materials and Methods

### 4.1. Plant Material

A total of 274 controlled crosses between early ripening peach, nectarine, flat peach, or flat nectarine accessions, all belonging to *Prunus persica*, and apricot, *Prunus armeniaca*, were used in this study, resulting in the harvest of 63,549 fruits during eight consecutive seasons, from 2014 to 2021 (Table 1). Most of the *Prunus persica* fruits were derived from the IRTA’s breeding program developed at the experimental orchard located at Gimenells, northeastern Spain. Some other *Prunus persica* fruits were provided by the private breeding company Vif International (Albalate de Cinca, Spain). The *Prunus armeniaca* fruits were provided by the private breeding company Newcot (Saint-Gilles, France). Fruits from each cross were harvested 10 days before commercial ripening, transported and stored in conventional cold rooms (1 to 3C) at the IRTA Fruitcentre, until embryo dissection was performed, from 1 to 4 weeks after reception. The in vitro germination efficiencies were recorded for all 274 crosses (Table 1), and for a subset of 142 crosses (Table 2), the efficiencies were evaluated for in vitro germination, plantlet development, and acclimatization to greenhouse conditions.

### 4.2. Embryo Dissection and Culture

Peach and nectarine fruits were opened with the help of pruning scissors. Apricot fruit flesh was removed with a knife and the kernel was open with a nutcracker. Seeds were extracted from the kernel, with the help of forceps if required, trying not to damage either the seed coat or the embryos. To reduce the microbial contaminations, seeds with teguments, in groups of 10 seeds, were disinfected with 1% NaClO for 10 min under shaking, followed by three rinses in sterile distilled water. Seeds were dissected under the naked eye or with the help of a dissecting scope, under aseptic conditions in a laminar flow hood, removing the seed coat and endosperm, trying to keep the embryo (cotyledons, plumule and radicle) as intact as possible. Each embryo’s maximum length, in mm, was measured by the naked eye with the help of an iron steel ruler, sterilized by heat and placed next to the dissecting surface. After that, the embryos were cultured in different types of culture vessels and media formulations, depending on the embryo size.

### 4.3. Culture Media

All the embryos were cultured in a basal medium composed of woody plant medium (WPM) [61] basal salts and vitamins (Duchefa Biochemie B.V., 2003 RV Haarlem, The Netherlands), supplemented with 30 g/L sucrose and 9 g/L agar (Agar E406, Quimivita, Barcelona, Spain), adjusted to pH 5.7 before autoclaving (M1). For embryos bigger than 5 mm long, two different media were used, named M1 and M1V. Both were based on the basal medium, and M1V was amended with 125% vermiculite (50/40, *v*/*v*, vermiculite/semisolid medium). Vermiculite (Termita-3, Castillo Arnedo, Calahorra, Spain) was dispensed into the culture vessels before the semisolid basal medium was added. Embryos smaller than 5 mm long at maximum length were cultured in M2 medium. This medium was the basal medium supplemented with 1 µM gibberellic acid (GA3) and 1 µM 6-benzylaminopurine (BAP) (Duchefa Biochemie B.V., 2003 RV Haarlem, the Netherlands). BAP was added to the medium before autoclaving, while GA3 was dissolved in dimethyl sulfoxide (DMSO) and added to the medium after its sterilization and cooling to 50 °C. The final DMSO concentration was 0.05% (*v*/*v*, DMSO/medium). M1 and M1V, once in the following culture vessels, were sterilized for 20 min at 121 °C, while M2 was sterilized in bottles and dispensed to Petri dishes after adding the GA3. The M2 medium was not assayed with vermiculite, because embryos smaller than 5 mm long were fragile and friable, would break when introduced in the culture medium, and had a better contact with the nutritious medium when cultured in semisolid medium with only agar.

### 4.4. Containers Used for Embryo Rescue

**Figure 6:**
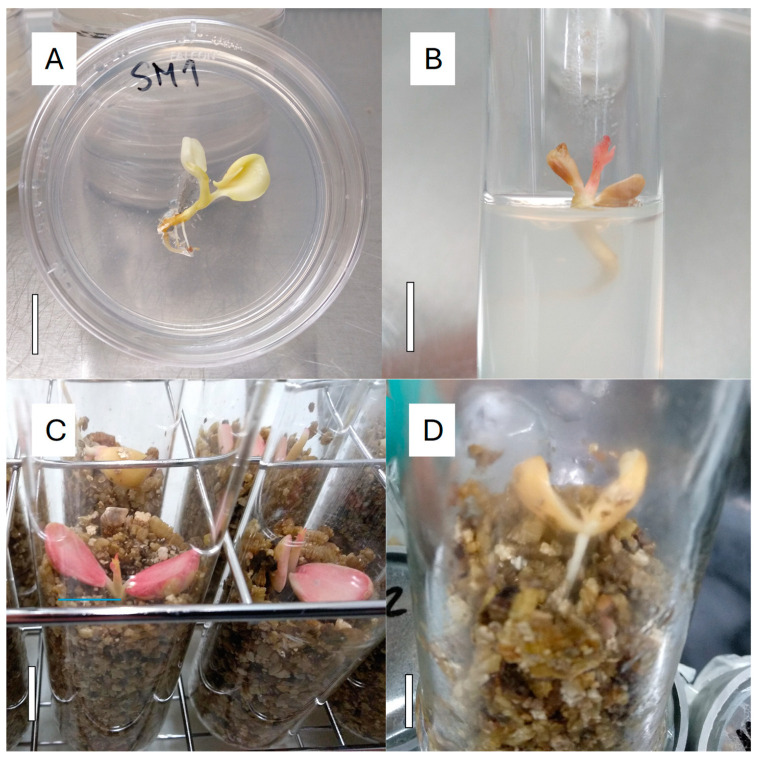
In vitro germinated immature *Prunus* spp. embryos in the control post stratification, at 4 °C, in (**A**) 60 mm wide Petri dishes, (**B**) 24 mm wide tubes, (**C**) 38 mm wide tubes, and (**D**) 42 mm glass jars. The white solid bar in each picture is equivalent to 1 cm in length.

**Figure 7:**
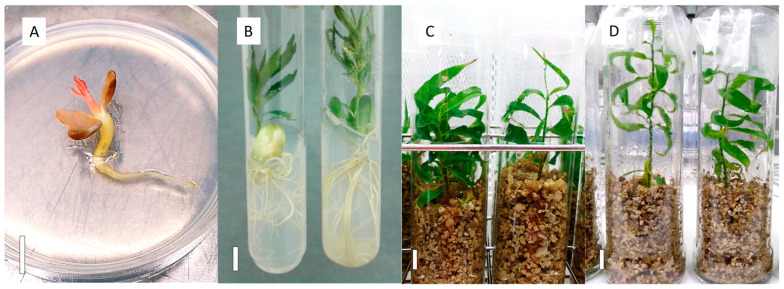
*Prunus* spp. plantlets from embryos grown at (**A**) 14 °C in Petri dishes containing M2, and at 24 °C in (**B**) 24 mm wide tubes containing M1 medium, in (**C**) 38 mm wide tubes containing M1V medium, and in (**D**) 42 mm glass jars containing M1V medium. The white solid bar in each picture is equivalent to 1 cm in length.

Embryos bigger than 5 mm long were cultured in either (1) glass tubes 38 mm wide and 160 mm high with an aerated polypropylene cap (Ref L10568, LineaLAB, Badalona, Spain) (Figure 6C and Figure 7C) or (2) glass jars 42 mm wide and 170 mm high (Ref 7cyl TO48, Apiglass, Barcelona, Spain) closed with a plastic layer P30PXNP (Ref PET 30 Melinex, ILPRA Systems S.L., Mataró, Spain) (Figure 6D and Figure 7D). This film is autoclavable, transparent, and with an oxygen gas transmission rate < 110 cm^3^/m^2^ day. A square of 10 × 10 cm was cut and fixed at the mouth of the jar with a PVC ring (47 mm internal diameter and 20 mm height). Each glass tube or jar contained 90 mL of M1V medium, and one embryo was cultured in each vessel. Embryos with a size bigger than 5 mm long were also individually cultured in glass tubes 24 mm wide and 150 mm high with aerated polypropylene caps (Ref N-51A KIMAX, Scharlab, S.L., Barcelona, Spain) (Figure 6B and Figure 7B), each containing 20 mL of M1 medium. Petri dishes, 60 mm wide and 15 mm high (Ref 82.1194.500, Sarstedt, La Roca del Vallès, Spain) (Figure 6A and Figure 7A), each containing 10 mL of M2 medium, were used to culture embryos smaller than 5 mm long, one in each dish.

### 4.5. Cold Stratification and Induction of Embryo Germination

The Petri dishes, racks of tubes or jars with embryos were wrapped with plastic film, placed inside dark plastic bags, and cultured in a dark, cold chamber set at 4 °C, for the 3-month-long stratification period. After this time, a first germination control was performed, recording (1) contaminated cultures, which were eliminated, (2) the number of clean embryos (C), and (3) the number of germinated embryos (G). Embryos started their germination when their cotyledons were unfolded and the embryo axis started to elongate, as shown in Figure 6. Non-germinated embryos were brought back to the cold culture room at 4 °C for 2 additional months. At this time, a second germination control was performed, recording the same parameters (C and G). These values were added to the values recorded in the first germination control. The ratio G/C, expressed in a percentage, was calculated for each cross (Table 1 and Table 2).

### 4.6. Plantlet Growth till Acclimatization

The germinated embryos after the first control and all the embryos after the second control were moved to an in vitro culture chamber at 14 °C and 150–200 µmol m^−2^ s^−1^ photosynthetically active radiation (PAR) of cool white, fluorescent light (Master TL5 HO 39W/840, Phillips, Polland), with a 12/12 h photoperiod, for a 1-month-long culture period. In the case of smaller embryos, which were germinated in Petri dishes (Figure 7A), they were transplanted to 24 mm wide and 150 mm high tubes with M1 medium to increase the available space for their development under these culture conditions. One month later and depending on the plantlet development, the tubes and flasks with plantlets (Figure 7B–D) were moved to a culture chamber at 24 °C, and 150–200 µmol m^−2^ s^−1^ of light intensity with a 16/8 h photoperiod, to accelerate the plantlet growth. The embryos rescued in M1V medium (Figure 7C,D), when necessary, were supplemented with 20 mL of liquid PRU0 medium in each vessel. This medium was half WPM basal salts and vitamins, supplemented with 50 µM FeEDTA, 6 µM CuSO_4_·5H_2_O, 30 g/L sucrose, adjusted to pH 5.7 before autoclaving. From 5- to 7-months post embryo dissection and culture, the number of developed plantlets (P), such as those in Figure 7B–D, was recorded for each cross.

### 4.7. Acclimatization to Greenhouse Conditions

**Figure 8:**
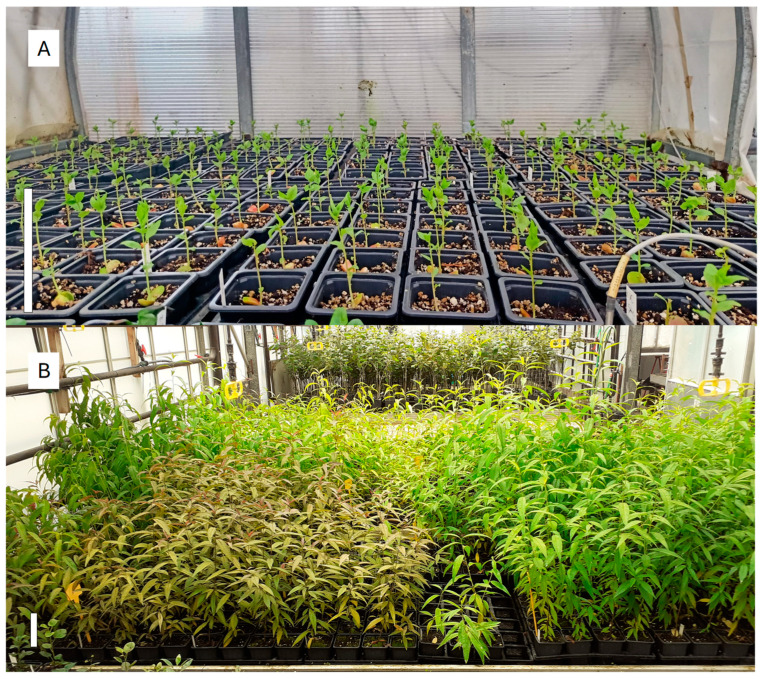
*Prunus* spp. plantlets derived from embryo rescue during (**A**) the initial phase of acclimatization to soil and (**B**) after hardening in the greenhouse and ready to be transferred to the field. The white solid bars in each picture are equivalent to 10 cm in length.

Plantlets with good shoot and root development (Figure 7B–D) were removed from the containers, cleaned up with tap water and planted in trays containing a peat:vermiculite mix substrate (2:1, *v*/*v*). The vermiculite was the same as used in the M1V medium. The peat substrate was the Exclusive (Gebr.Brill Substrate GmbH & Co. KG, Georgsdorf, Germany). The plants were acclimated in plastic tunnels (Figure 8A), within a conventional greenhouse, designed to decrease the atmospheric relative humidity gradually and automatically, from 100 to 60%, during a 3- to 4-week-long period. The acclimatization tunnels had a soil temperature above 22/18 °C (day/night) and a photoperiod of 16 h light, supplemented with LED lights (SUP12100DC, AlternativaLED, Terrassa, Barcelona, Spain) to extend the daylight hours, at 230 μmol m^−2^ s^−1^ PAR at leaf level. After this period, the trays were removed from the tunnels and cultured in a greenhouse for their hardening, watered by a mist system until they were hard enough to be transferred into an evaluation plot. At this point (Figure 8B), the number of acclimated plants (A) was determined for each cross (Table 2). A total of 6260 plants derived from the M1 medium (1375 apricots, 1795 nectarines with white flesh, 2269 nectarines with yellow flesh, 8 peaches non-melting, 564 peaches with white flesh, and 249 peaches with yellow flesh) and 2941 from the M1V medium (1088 apricots, 1090 nectarines with white flesh, 530 nectarines with yellow flesh, 22 peaches non-melting, 199 peaches with white flesh, and 13 peaches with yellow flesh) were brought through the acclimatization process. A total of 5109 and 2330 acclimated plants were produced from M1 and M1V, respectively.

### 4.8. Statistical Data Analysis

The experiments were design considering a completely random design, and the data analysis was carried out by using JMP Pro software (version 17.0.0, JMP Statistical Discovery LLC, Cary, NC, USA). Data concerning each measured variable were analyzed with ANOVA using the JMP’s “Fit Model” and “Fit Y by X” scripts, considering the fruit and flesh type, embryo size, or culture media as factors. Each directed cross within the same fruit type, as indicated in Table 1 and Table 2, was considered a replicate for each measured variable. The percentages of germination over uncontaminated embryos in Figure 2 and Figure 3 were measured for the crosses indicated in Table 1, and those in Figure 5A from the crosses indicated Table 2. The percentages of acclimated plants over in vitro grown plantlets (Figure 5B) and percentages of acclimated plants over uncontaminated embryos (Figure 5C) were measured for the crosses indicated in Table 2. JMP’s outputs are included in Supplementary Materials S1 for Figure 2, Supplementary Materials S2 for Figure 3, and Supplementary Materials S3 for Figure 5. Statistical significance was judged at *p* ≤ 0.05, and Tukey’s HSD test was used for the mean separation when the differences were statistically significant.

## 5. Conclusions

The *Prunus* spp. immature embryo rescue efficiencies, as viable plants transferred to field plots over uncontaminated embryos, primarily depend on the fruit type, embryo size, fruit quality at harvest and conservation prior the seed dissection out of the pericarp. Endo- and epiphytic embryo contamination must be reduced or controlled to improve the rescue of viable plants. In addition, embryo germination after culture at 4 °C and acclimatization of plantlets to soil are the main variables determining the differential embryo rescue efficiencies for the different fruit types. The overall efficiencies of the protocol described herein run from 8 to 23% acclimated plants over uncontaminated embryos for flat peaches and flat nectarines, 31 to 54% for peaches, 49 to 56% for nectarines, and 46% for apricots.

The use of vermiculite in the culture medium, compared to agar-containing medium, had no detrimental effects on the in vitro embryo germination and was beneficial for the development of the rhizosphere, stem, and foliar area, which was favorable for the plantlet acclimatization to soil. Affordable glass containers, as used in the food industry, and the use of gas-permeable films to close the flask, make the protocol described herein a feasible and reliable methodology for *Prunus* spp. breeding programs in which the manipulation of thousands of embryos is necessary.

## Figures and Tables

**Table 1:** Total number of directed crosses, fruits and rescued embryos used in the present work.

Species	Fruit Type	Flesh Type	Crosses	Fruits	Embryos
*Prunus persica* (L.) Batsch	Peach	Yellow	18	2778	2838
*Prunus persica* (L.) Batsch	Peach	White	16	3984	3469
*Prunus persica* (L.) Batsch	Peach	Non-melting (Pavia)	8	434	391
*Prunus persica* (L.) Batsch	Peach	Stony Hard (Albino)	3	717	660
*Prunus persica* (L.) Batsch	Flat Peach	Yellow	7	1350	1341
*Prunus persica* (L.) Batsch	Flat Peach	White	65	10,792	10,553
*Prunus persica* (L.) Batsch	*Nectarine*	Yellow	53	20,121	18,329
*Prunus persica* (L.) Batsch	*Nectarine*	White	47	14,684	12,555
*Prunus persica* (L.) Batsch	Flat Nectarine	White	14	4842	2009
*Prunus armeniaca*	Apricot	*-*	43	3847	3811
Total			274	63,549	55,956

**Table 2:** Crosses used in the analysis of the in vitro plantlet development and greenhouse acclimatization.

Species	Fruit Type	Flesh Type	Crosses	Fruits	Embryos
*Prunus persica* (L.) Batsch	Peach	Yellow	6	1430	1289
*Prunus persica* (L.) Batsch	Peach	White	9	2234	1990
*Prunus persica* (L.) Batsch	Peach	Non-melting (Pavia)	3	93	89
*Prunus persica* (L.) Batsch	Flat Peach	Yellow	3	370	348
*Prunus persica* (L.) Batsch	Flat Peach	White	26	5089	4567
*Prunus persica* (L.) Batsch	*Nectarine*	Yellow	20	8053	7054
*Prunus persica* (L.) Batsch	*Nectarine*	White	27	7524	6517
*Prunus persica* (L.) Batsch	Flat Nectarine	White	5	989	799
*Prunus armeniaca*	Apricot	*-*	43	3847	3811
Total			142	29,629	26,464

## Data Availability

The data presented in this study are available on request from the corresponding author. Access to plant material names or identifications is restricted due to the private breeding programs’ interests.

## Supplementary Materials

The following supporting information can be downloaded at https://www.mdpi.com/article/10.3390/plants13212953/s1, Statistical outputs form JMP giving support to the separation of means shown in Figure 2, Figure 3, and Figure 5.
