# Supplementary material for: Development of a Feasible and Efficient In Vitro Rescue Protocol for Immature Prunus spp. Embryos
Source: Plants (Basel). 2024 Oct 22;13(21):2953. doi: 10.3390/plants13212953 (PMC11547775; doi:10.3390/plants13212953)
Supplement: Supplementary file 1 [file plants-13-02953-s001.zip › plants-3213712-supplementary.pdf]

Supplementary Materials - S1

Statistical outputs for Figure 2. Percentage of germinated over uncontaminated embryos, of sizes <5 mm or ≥5 mm, for different fruit and flesh types.

Software used.

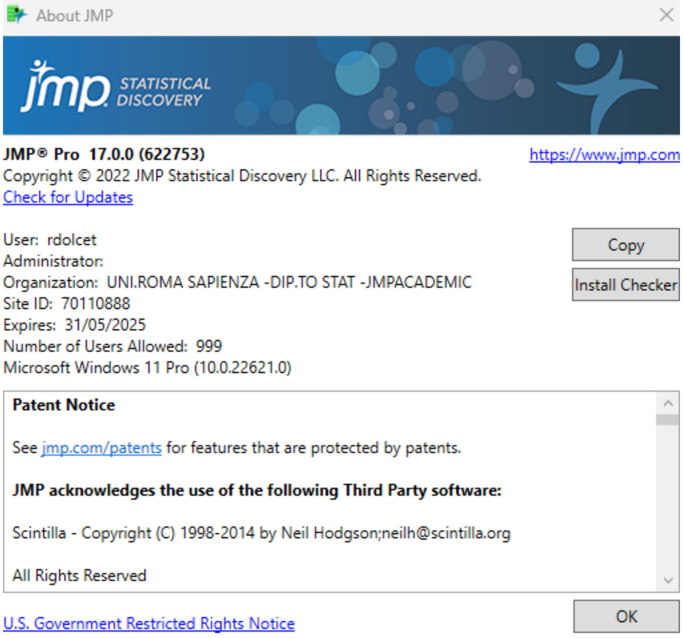

Analyze Fit Model

Factors: Fruit and Flesh Types, Embryo Size.
Variable: Germinated over Uncontaminated Embryos.

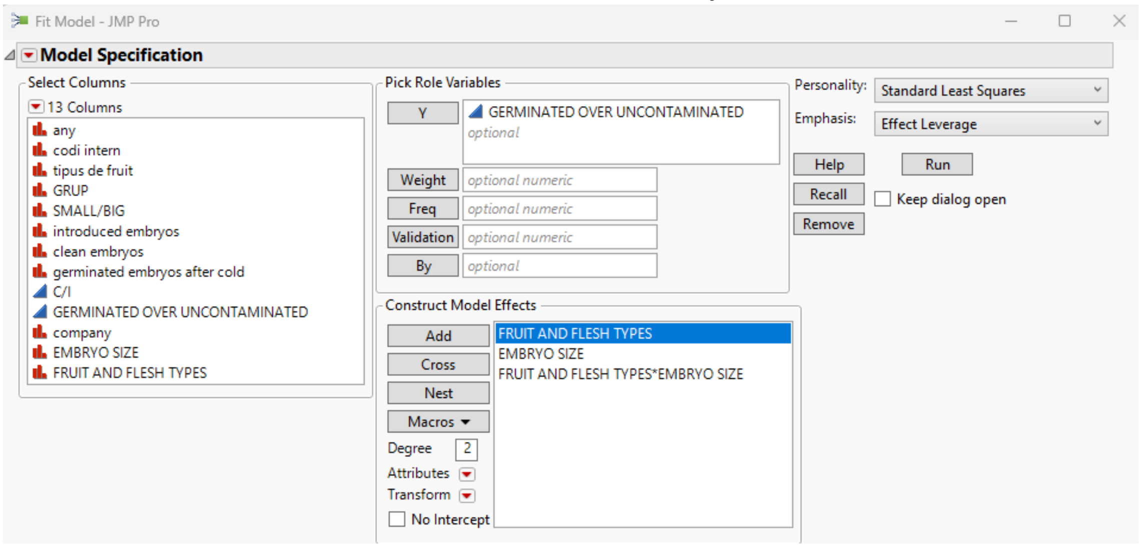

Response GERMINATED OVER UNCONTAMINATED

Singularity Details

Term	Details
FRUIT AND FLESH TYPES[PEACH-NON-MELTING]	=FRUIT AND FLESH TYPES[PEACH-STONY HARD] + FRUIT AND FLESH T...

Whole Model

Actual by Predicted Plot

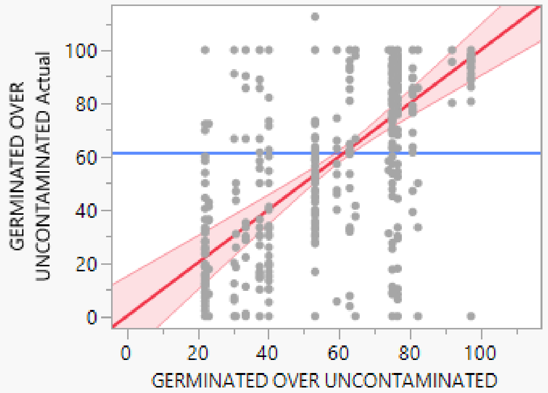

Predicted RMSE=26,676 RSq=0,47 PValue=<,0001

Effect Summary

Source	Logworth		PValue
FRUIT AND FLESH TYPES	22,673		0,00000
FRUIT AND FLESH TYPES*EMBRYO SIZE	0,713		0,19355
EMBRYO SIZE	.		.

Remove Add Edit ☐ FDR

There is no significant interaction between both factors (p=0.1936)

There is a significant effect of fruit and flesh types (p<0.0001)

Whole Model

Residual by Predicted Plot

Summary of Fit

Analysis of Variance

Source	DF	Sum of Squares	Mean Square	F Ratio
Model	17	340157,23	20009,2	28,1186
Error	539	383552,92	711,6	Prob > F
C. Total	556	723710,15		<,0001*

Parameter Estimates

Term		Estimate	Std Error	t Ratio	Prob>|t|
Intercept	Biased	48,322883	5,323152	9,08	<,0001*
FRUIT AND FLESH TYPES[APRICOT]	Biased	41,430604	6,580775	6,30	<,0001*
FRUIT AND FLESH TYPES[FLAT NECTARINE-WHITE]	Biased	-7,075784	7,362903	-0,96	0,3370
FRUIT AND FLESH TYPES[FLAT PEACH-WHITE]	Biased	-10,67931	5,738013	-1,86	0,0633
FRUIT AND FLESH TYPES[FLAT PEACH-YELLOW]	Biased	4,0856136	8,458149	0,48	0,6293
FRUIT AND FLESH TYPES[NECTARINE-WHITE]	Biased	7,9487743	5,917089	1,34	0,1797
FRUIT AND FLESH TYPES[NECTARINE-YELLOW]	Biased	9,9910475	5,836198	1,71	0,0875
FRUIT AND FLESH TYPES[PEACH-NON-MELTING]	Biased	-71,20383	38,0528	-1,87	0,0619
FRUIT AND FLESH TYPES[PEACH-STONY HARD]	Biased	18,363049	13,98804	1,31	0,1898
FRUIT AND FLESH TYPES[PEACH-WHITE]	Biased	7,1890299	5,536821	1,30	0,1947
EMBRYO SIZE[BIG]	Biased	25,227704	5,052209	4,99	<,0001*
FRUIT AND FLESH TYPES[APRICOT]*EMBRYO SIZE[BIG]	Biased	-17,70865	6,430616	-2,75	0,0061*
FRUIT AND FLESH TYPES[FLAT NECTARINE-WHITE]*EMBRYO SIZE[BIG]	Biased	-7,132872	7,607254	-0,94	0,3488
FRUIT AND FLESH TYPES[FLAT PEACH-WHITE]*EMBRYO SIZE[BIG]	Biased	-9,684542	5,596407	-1,73	0,0841
FRUIT AND FLESH TYPES[FLAT PEACH-YELLOW]*EMBRYO SIZE[BIG]	Biased	-3,596373	8,977147	-0,40	0,6889
FRUIT AND FLESH TYPES[NECTARINE-WHITE]*EMBRYO SIZE[BIG]	Biased	-6,499711	5,7674	-1,13	0,2603
FRUIT AND FLESH TYPES[NECTARINE-YELLOW]*EMBRYO SIZE[BIG]	Biased	-7,048798	5,672234	-1,24	0,2145
FRUIT AND FLESH TYPES[PEACH-NON-MELTING]*EMBRYO SIZE[BIG]	Biased	62,209867	37,34257	1,67	0,0963
FRUIT AND FLESH TYPES[PEACH-STONY HARD]*EMBRYO SIZE[BIG]	Zeroed	0	0	.	.
FRUIT AND FLESH TYPES[PEACH-WHITE]*EMBRYO SIZE[BIG]	Zeroed	0	0	.	.

Effect Tests

Source	Nparm	DF	Sum of Squares	F Ratio	Prob > F	
FRUIT AND FLESH TYPES	9	7	97533,532	19,5803	<,0001*	LostDFs
EMBRYO SIZE	1	0	0,000	.	.	LostDFs
FRUIT AND FLESH TYPES*EMBRYO SIZE	9	7	7086,416	1,4226	0,1935	LostDFs

For apricot there is a significantly different effect of embryo size on the variable (p=0.0061).

Analyze Fit Y by X

Factor: Embryo Size.
Variable: Germinated over Uncontaminated Embryos.

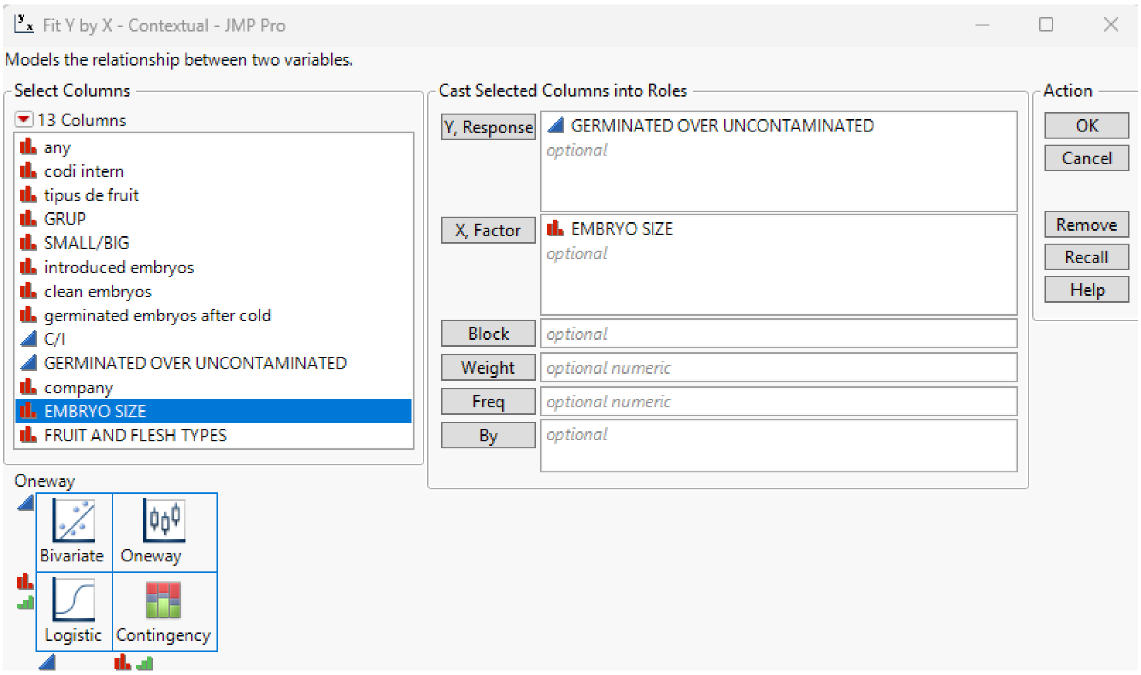

Oneway Analysis of GERMINATED OVER UNCONTAMINATED By EMBRYO SIZE

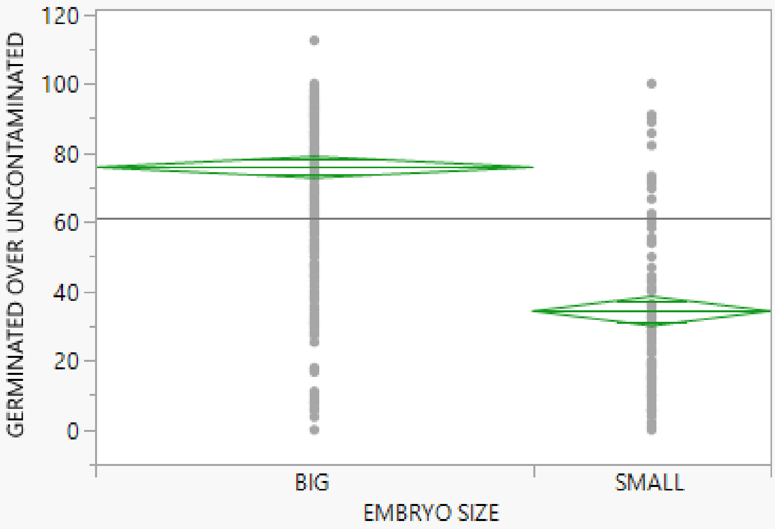

Oneway Anova

Summary of Fit

Pooled t Test

Analysis of Variance

Source	DF	Sum of Squares	Mean Square	F Ratio	Prob > F
EMBRYO SIZE	1	219668,06	219668	241,8762	<,0001*
Error	555	504042,09	908		
C. Total	556	723710,15			

Means for Oneway Anova

Level	Number	Mean	Std Error	Lower 95%	Upper 95%
BIG	361	75,8688	1,5861	72,753	78,984
SMALL	196	34,2845	2,1526	30,056	38,513

Std Error uses a pooled estimate of error variance

Missing Rows	6
Excluded Rows	1

There is a significant effect of embryo size on the variable ($p<0.0001$)

Analyze Fit Y by X:

Factor: Fruit and Flesh Types.
Variable: Germinated over Uncontaminated Embryos.

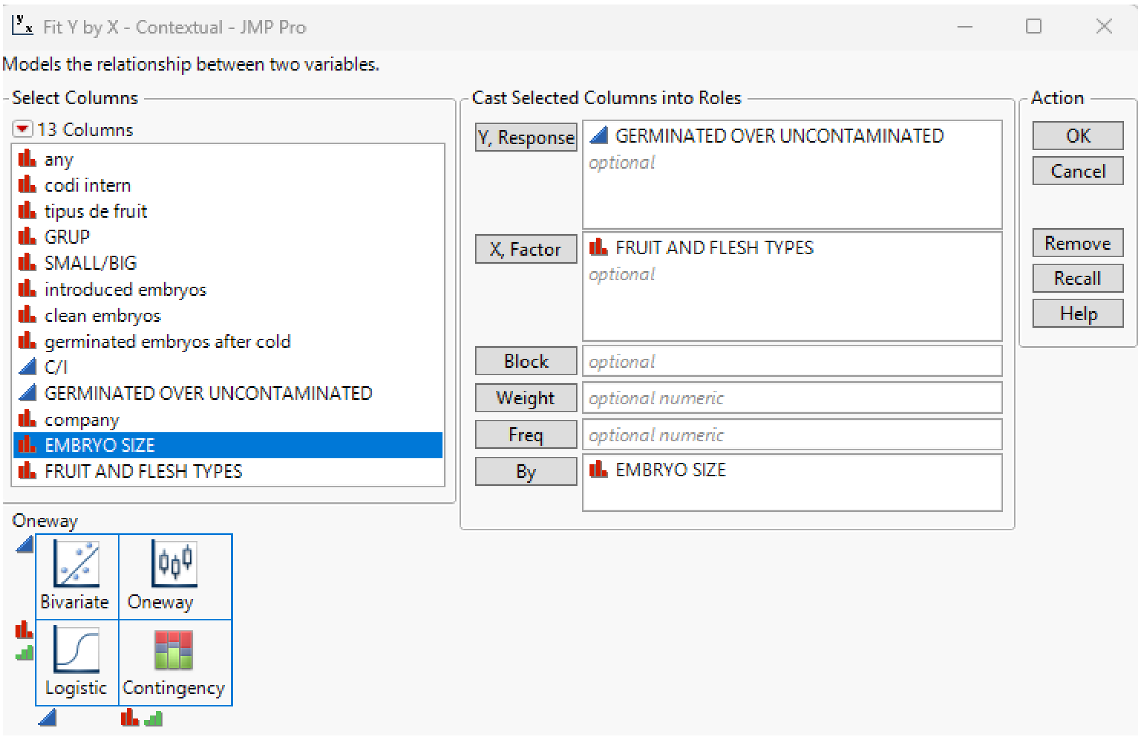

Oneway Analysis of GERMINATED OVER UNCONTAMINATED By FRUIT AND FLESH TYPES EMBRYO SIZE=BIG

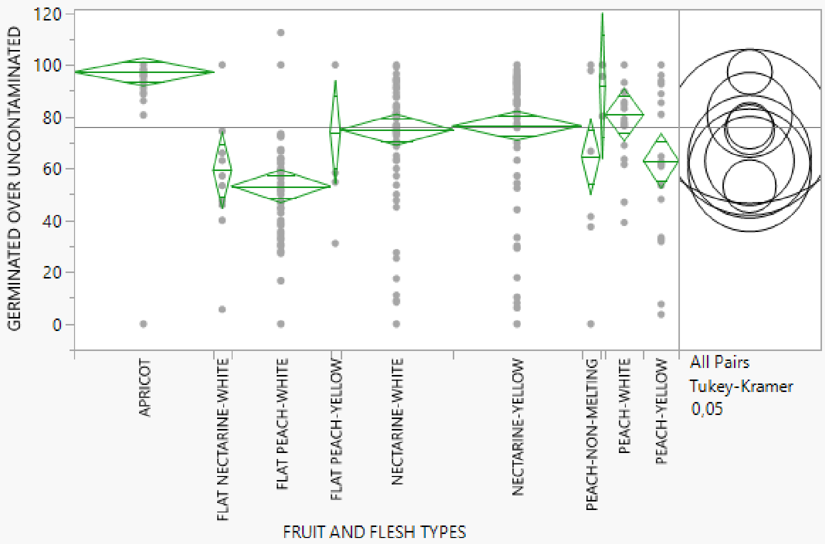

Oneway Anova

Summary of Fit

Analysis of Variance

Source	DF	Sum of Squares	Mean Square	F Ratio	Prob > F
FRUIT AND FLESH TYPES	9	77706,88	8634,10	14,1514	<,0001*
Error	351	214152,53	610,12		
C. Total	360	291859,41			

There are significant differences between Fruit and Flesh Types for big embryos (p<0.0001).

Oneway Anova

Means for Oneway Anova

Level	Number	Mean	Std Error	Lower 95%	Upper 95%
APRICOT	83	97,2725	2,711	91,940	102,60
FLAT NECTARINE-WHITE	11	59,3419	7,448	44,695	73,99
FLAT PEACH-WHITE	59	53,1867	3,216	46,862	59,51
FLAT PEACH-YELLOW	6	74,0398	10,084	54,207	93,87
NECTARINE-WHITE	67	74,9997	3,018	69,065	80,93
NECTARINE-YELLOW	77	76,4928	2,815	70,957	82,03
PEACH-NON-MELTING	11	64,5566	7,448	49,909	79,20
PEACH-STONY HARD	3	91,9136	14,261	63,866	119,96
PEACH-WHITE	23	80,7396	5,150	70,610	90,87
PEACH-YELLOW	21	62,9625	5,390	52,361	73,56

Std Error uses a pooled estimate of error variance

Means Comparisons

Comparisons for all pairs using Tukey-Kramer HSD

Confidence Quantile

q*	Alpha
3,18422	0,05

HSD Threshold Matrix

Connecting Letters Report

Level				Mean
APRICOT	A			97,272543
PEACH-STONY HARD	A	B	C	91,913636
PEACH-WHITE	A	B		80,739617
NECTARINE-YELLOW		B		76,492836
NECTARINE-WHITE		B		74,999651
FLAT PEACH-YELLOW	A	B	C	74,039827
PEACH-NON-MELTING		B	C	64,556621
PEACH-YELLOW		B	C	62,962473
FLAT NECTARINE-WHITE		B	C	59,341931
FLAT PEACH-WHITE			C	53,186736

Levels not connected by same letter are significantly different.

Separation of means according to Tuckey-Kramer HSD (p=0.05) between Fruit and Flesh Types, for big embryos.

Oneway Analysis of GERMINATED OVER UNCONTAMINATED By FRUIT AND FLESH TYPES EMBRYO SIZE=SMALL

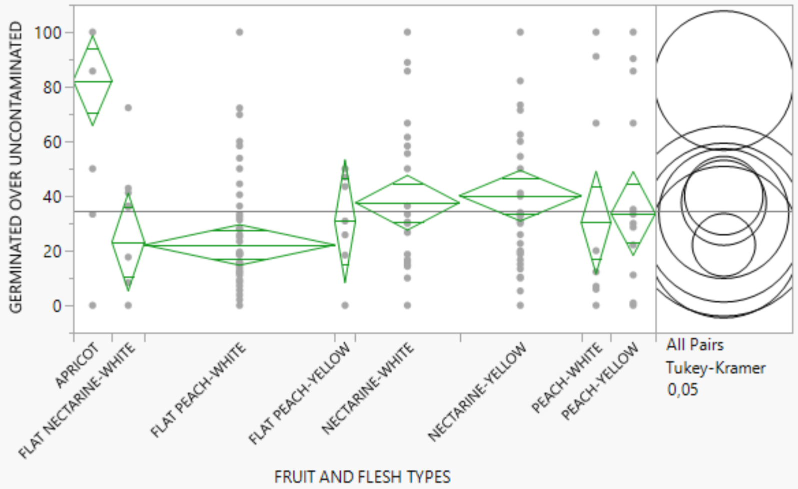

Oneway Anova

Summary of Fit

Analysis of Variance

Source	DF	Sum of Squares	Mean Square	F Ratio	Prob > F
FRUIT AND FLESH TYPES	7	42782,29	6111,76	6,7828	<,0001*
Error	188	169400,39	901,07		
C. Total	195	212182,68			

There are significant differences between Fruit and Flesh Types for small embryos (p<0.0001).

Oneway Anova

Analysis of Variance

Source	DF	Sum of Squares	Mean Square	F Ratio	Prob > F
FRUIT AND FLESH TYPES	7	42782,29	6111,76	6,7828	<,0001*
Error	188	169400,39	901,07		
C. Total	195	212182,68			

Means for Oneway Anova

Level	Number	Mean	Std Error	Lower 95%	Upper 95%
APRICOT	13	82,2344	8,325	65,811	98,658
FLAT NECTARINE-WHITE	11	23,1523	9,051	5,298	41,006
FLAT PEACH-WHITE	64	22,1004	3,752	14,699	29,502
FLAT PEACH-YELLOW	7	30,7772	11,346	8,396	53,158
NECTARINE-WHITE	35	37,5437	5,074	27,535	47,553
NECTARINE-YELLOW	41	40,1350	4,688	30,887	49,383
PEACH-WHITE	10	30,2842	9,492	11,559	49,010
PEACH-YELLOW	15	33,5849	7,751	18,296	48,874

Std Error uses a pooled estimate of error variance

Means Comparisons

Comparisons for all pairs using Tukey-Kramer HSD

Confidence Quantile

q*	Alpha
3,06539	0,05

HSD Threshold Matrix

Connecting Letters Report

Level			Mean
APRICOT	A		82,234432
NECTARINE-YELLOW		B	40,135025
NECTARINE-WHITE		B	37,543664
PEACH-YELLOW		B	33,584908
FLAT PEACH-YELLOW		B	30,777166
PEACH-WHITE		B	30,284209
FLAT NECTARINE-WHITE		B	23,152267
FLAT PEACH-WHITE		B	22,100412

Levels not connected by same letter are significantly different.

Separation of means according to Tuckey-Kramer HSD (p=0.05) between Fruit and Flesh Types, for small embryos.

Supplementary Materials – S2

Statistical outputs for Figure 3. Percentage of germinated over uncontaminated embryos, for different fruit types, cultured in M1 or M1V media.

Software used.

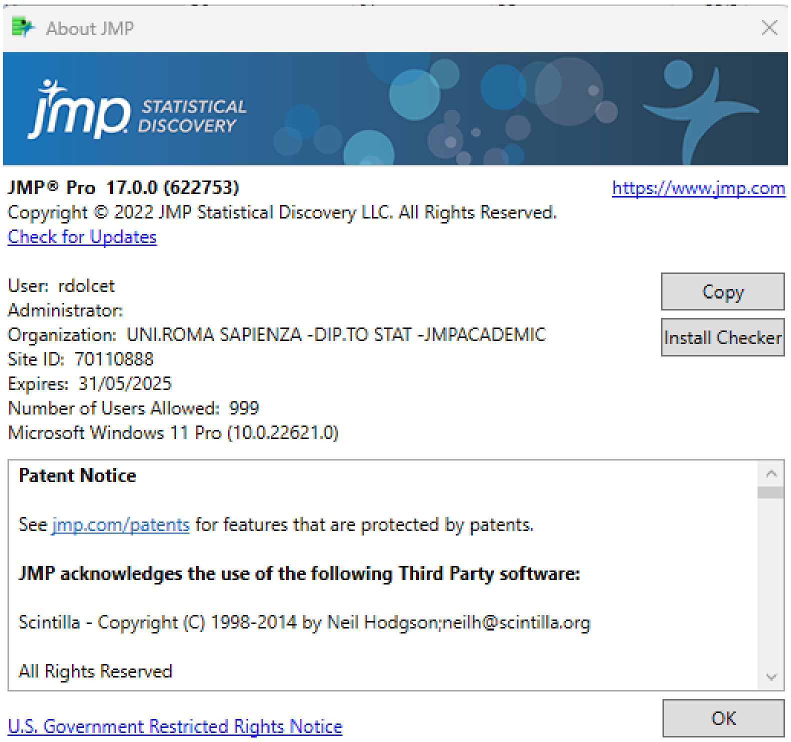

Analyze Fit Model

Factors: Fruit and Flesh Types, Medium (M1, M1V).
Variable: Germinated over Uncontaminated Embryos.

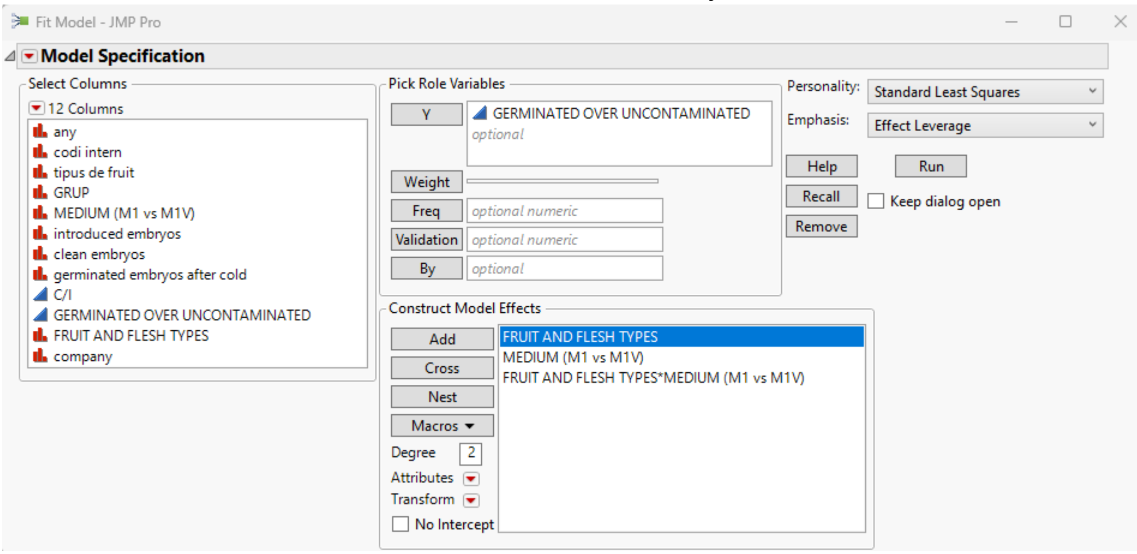

Response GERMINATED OVER UNCONTAMINATED

Whole Model

Actual by Predicted Plot

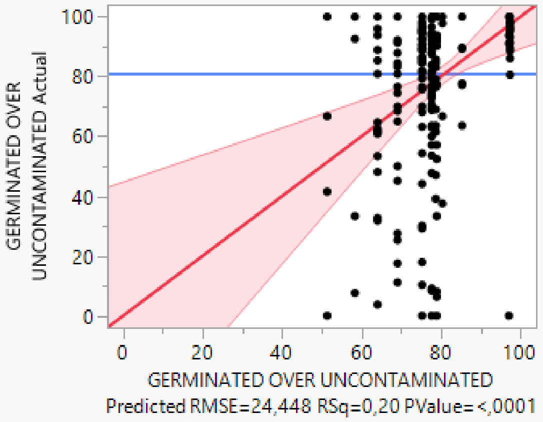

Effect Summary

Source	Logworth	PValue
FRUIT AND FLESH TYPES	9,153	0,00000
MEDIUM (M1 vs M1V)	0,727	0,18757
FRUIT AND FLESH TYPES*MEDIUM (M1 vs M1V)	0,524	0,29955

Remove Add Edit FDR

Residual by Predicted Plot

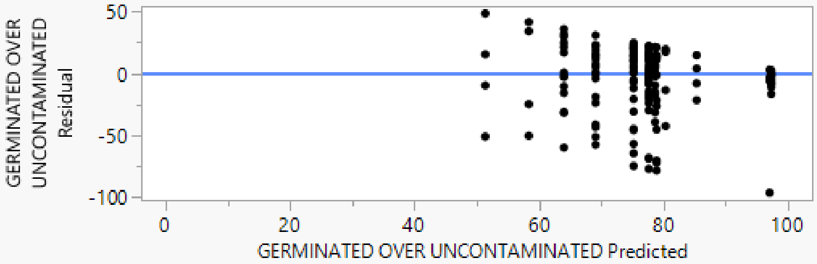

There is no significant interaction between both factors (p=0.2996)

There is a significant effect of fruit and flesh types (p<0.0001)

Summary of Fit

RSquare	0,197289
RSquare Adj	0,164586
Root Mean Square Error	24,44795
Mean of Response	81,12801
Observations (or Sum Wgts)	282

Analysis of Variance

Source	DF	Sum of Squares	Mean Square	F Ratio
Model	11	39663,60	3605,78	6,0327
Error	270	161379,63	597,70	**Prob > F**
C. Total	281	201043,23		<,0001*

Parameter Estimates

Term	Estimate	Std Error	t Ratio	Prob>|t|
Intercept	76,1484	2,094124	36,36	<,0001*
FRUIT AND FLESH TYPES[APRICOT]	21,116339	3,031906	6,96	<,0001*
FRUIT AND FLESH TYPES[NECTARINE-WHITE]	-2,750366	3,360746	-0,82	0,4139
FRUIT AND FLESH TYPES[NECTARINE-YELLOW]	0,9520906	3,189052	0,30	0,7655
FRUIT AND FLESH TYPES[PEACH- NON-MELTING]	-10,26507	6,396218	-1,60	0,1097
FRUIT AND FLESH TYPES[PEACH-WHITE]	5,8873147	4,984218	1,18	0,2386
MEDIUM (M1 vs M1V)[M1]	2,7666734	2,094124	1,32	0,1876
FRUIT AND FLESH TYPES[APRICOT]*MEDIUM (M1 vs M1V)[M1]	-2,624435	3,031906	-0,87	0,3875
FRUIT AND FLESH TYPES[NECTARINE-WHITE]*MEDIUM (M1 vs M1V)[M1]	1,5265978	3,360746	0,45	0,6500
FRUIT AND FLESH TYPES[NECTARINE-YELLOW]*MEDIUM (M1 vs M1V)[M1]	-4,616183	3,189052	-1,45	0,1489
FRUIT AND FLESH TYPES[PEACH- NON-MELTING]*MEDIUM (M1 vs M1V)[M1]	11,749993	6,396218	1,84	0,0673
FRUIT AND FLESH TYPES[PEACH-WHITE]*MEDIUM (M1 vs M1V)[M1]	-6,102388	4,984218	-1,22	0,2219

Effect Tests

Source	Nparm	DF	Sum of Squares	F Ratio	Prob > F
FRUIT AND FLESH TYPES	5	5	33667,999	11,2658	<,0001*
MEDIUM (M1 vs M1V)	1	1	1043,269	1,7455	0,1876
FRUIT AND FLESH TYPES*MEDIUM (M1 vs M1V)	5	5	3648,220	1,2207	0,2995

For apricot there is a significantly different effect on the variable ($p<0.001$).

Analize Fit Y by X

Factor: Fruit and Flesh Types.
Variable: Germinated over Uncontaminated Embryos.
Separated by: Medium (M1, M1V)

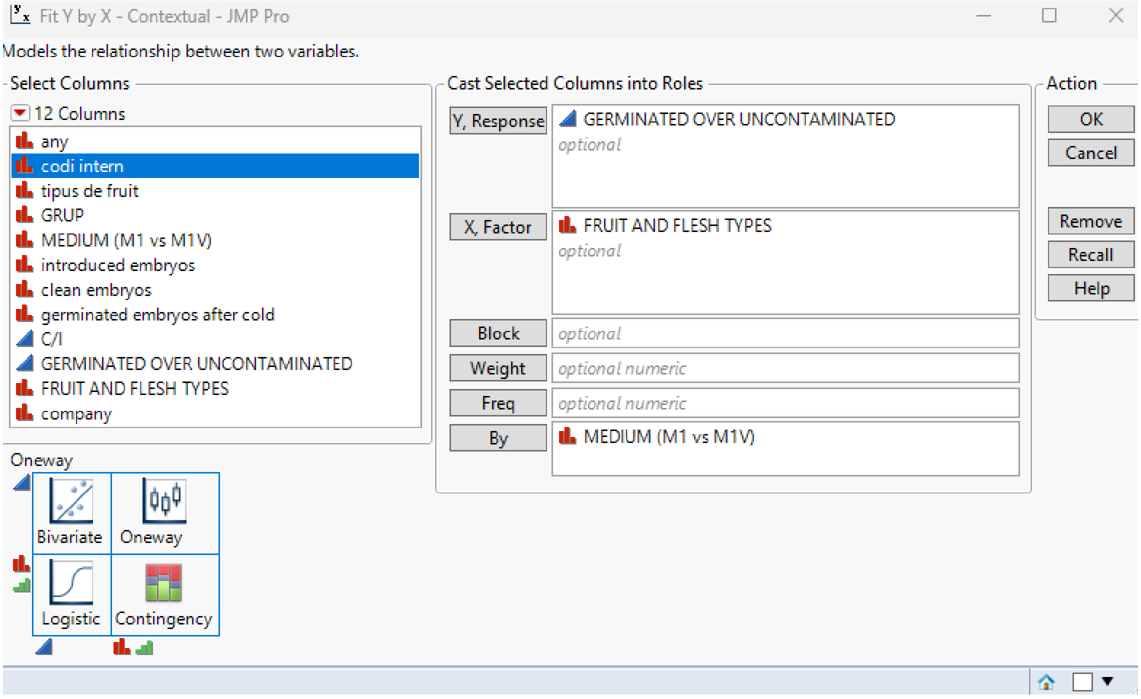

Oneway Analysis of GERMINATED OVER UNCONTAMINATED By FRUIT AND FLESH TYPES MEDIUM (M1 vs M1V)=M1

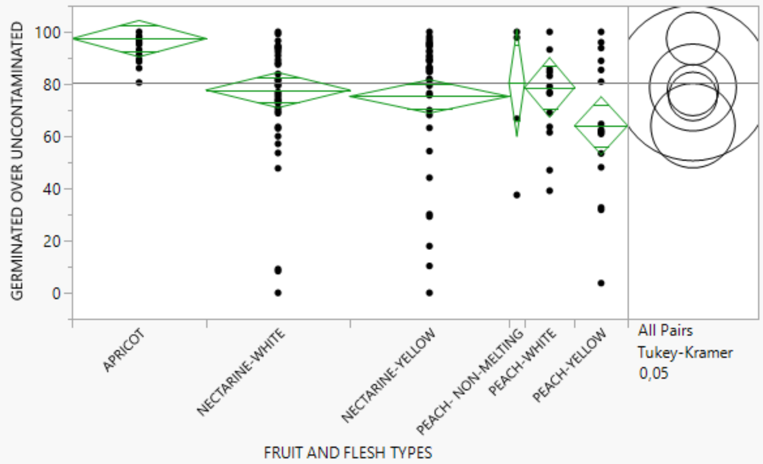

Oneway Anova

Summary of Fit

Analysis of Variance

Source	DF	Sum of Squares	Mean Square	F Ratio	Prob > F
FRUIT AND FLESH TYPES	5	18714,01	3742,80	7,0080	<,0001*
Error	172	91861,53	534,08		
C. Total	177	110575,54			

Means for Oneway Anova

Level	Number	Mean	Std Error	Lower 95%	Upper 95%
APRICOT	43	97,4070	3,524	90,451	104,36
NECTARINE-WHITE	46	77,6913	3,407	70,966	84,42
NECTARINE-YELLOW	51	75,2510	3,236	68,863	81,64
PEACH- NON-MELTING	5	80,4000	10,335	60,000	100,80
PEACH-WHITE	16	78,7000	5,778	67,296	90,10
PEACH-YELLOW	17	64,0412	5,605	52,978	75,10

Std Error uses a pooled estimate of error variance

There are significant differences among Fruit and Flesh Types for M1 medium (p<0.0001).

Means Comparisons

Comparisons for all pairs using Tukey-Kramer HSD

Confidence Quantile

q*	Alpha
2,88212	0,05

HSD Threshold Matrix

Connecting Letters Report

Level			Mean
APRICOT	A		97,406977
PEACH- NON-MELTING	A	B	80,400000
PEACH-WHITE	A	B	78,700000
NECTARINE-WHITE		B	77,691304
NECTARINE-YELLOW		B	75,250980
PEACH-YELLOW		B	64,041176

Levels not connected by same letter are significantly different.

Separation of means according to Tuckey-Kramer HSD (p=0.05) between Fruit and Flesh Types, for M1 medium.

Oneway Analysis of GERMINATED OVER UNCONTAMINATED By FRUIT AND FLESH TYPES MEDIUM (M1 vs M1V)=M1V

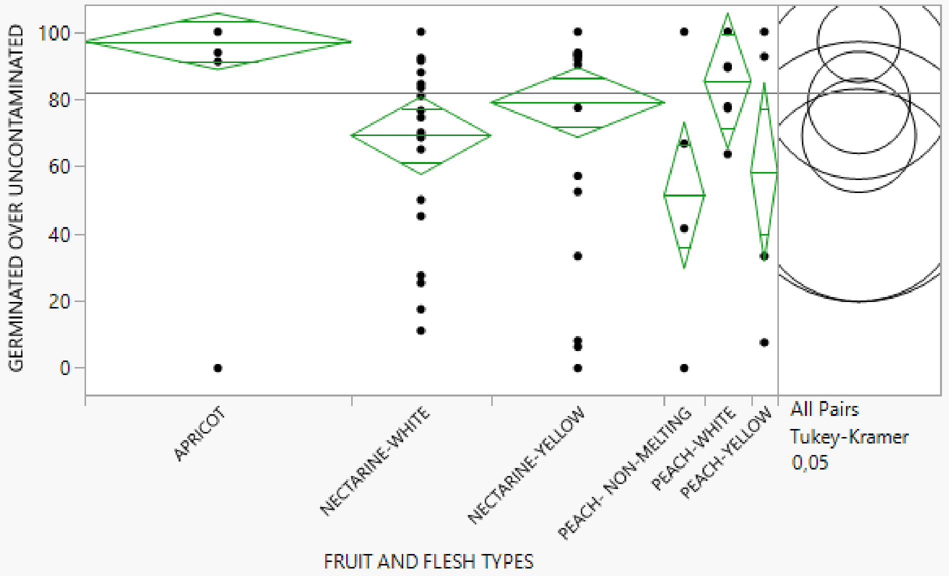

Oneway Anova

Summary of Fit

Analysis of Variance

Source	DF	Sum of Squares	Mean Square	F Ratio	Prob > F
FRUIT AND FLESH TYPES	5	20824,031	4164,81	5,8711	<,0001*
Error	98	69518,099	709,37		
C. Total	103	90342,130			

There are significant differences among Fruit and Flesh Types for M1V medium ($p<0.0001$).

Means for Oneway Anova

Level	Number	Mean	Std Error	Lower 95%	Upper 95%
APRICOT	40	97,1225	4,211	88,766	105,48
NECTARINE-WHITE	21	69,1048	5,812	57,571	80,64
NECTARINE-YELLOW	26	78,9500	5,223	68,584	89,32
PEACH- NON-MELTING	6	51,3667	10,873	29,789	72,94
PEACH-WHITE	7	85,3714	10,067	65,394	105,35
PEACH-YELLOW	4	58,3750	13,317	31,948	84,80

Std Error uses a pooled estimate of error variance

Means Comparisons

Comparisons for all pairs using Tukey-Kramer HSD

Confidence Quantile

q*	Alpha
2,90688	0,05

HSD Threshold Matrix

Connecting Letters Report

Level			Mean
APRICOT	A		97,122500
PEACH-WHITE	A	B	85,371429
NECTARINE-YELLOW	A	B	78,950000
NECTARINE-WHITE		B	69,104762
PEACH-YELLOW	A	B	58,375000
PEACH- NON-MELTING		B	51,366667

Levels not connected by same letter are significantly different.

Separation of means according to Tuckey-Kramer HSD (p=0.05) between Fruit and Flesh Types, for M1V medium.

Supplementary Materials – S3

Statistical outputs for Figure 5. Embryo rescue efficiencies in percentages of (A) in vitro germinated over uncontaminated embryos, (B) acclimated plants in the greenhouse over the in vitro developed plantlets, and (C) acclimated plants over uncontaminated embryos.

Software used.

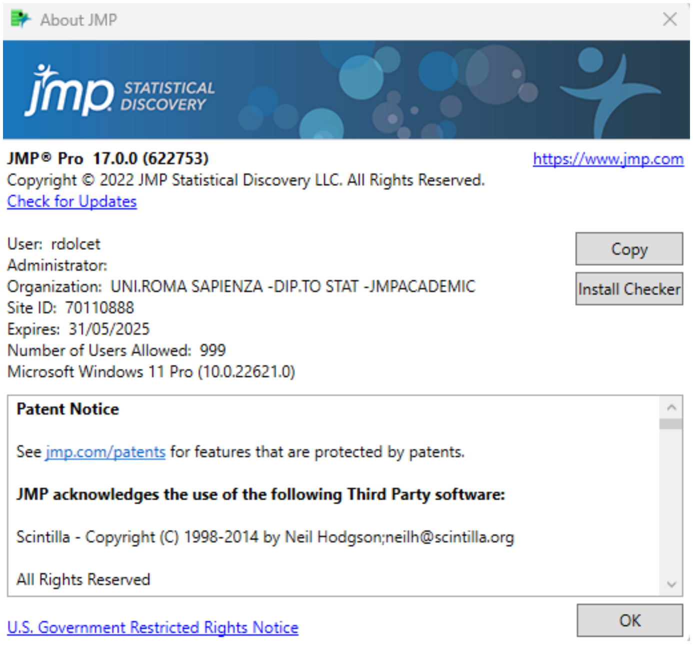

Figure 5A

Analyze Fit Y by X.

Factors: Fruit and Flesh Types.
Variable: Germinated over Uncontaminated Embryos.

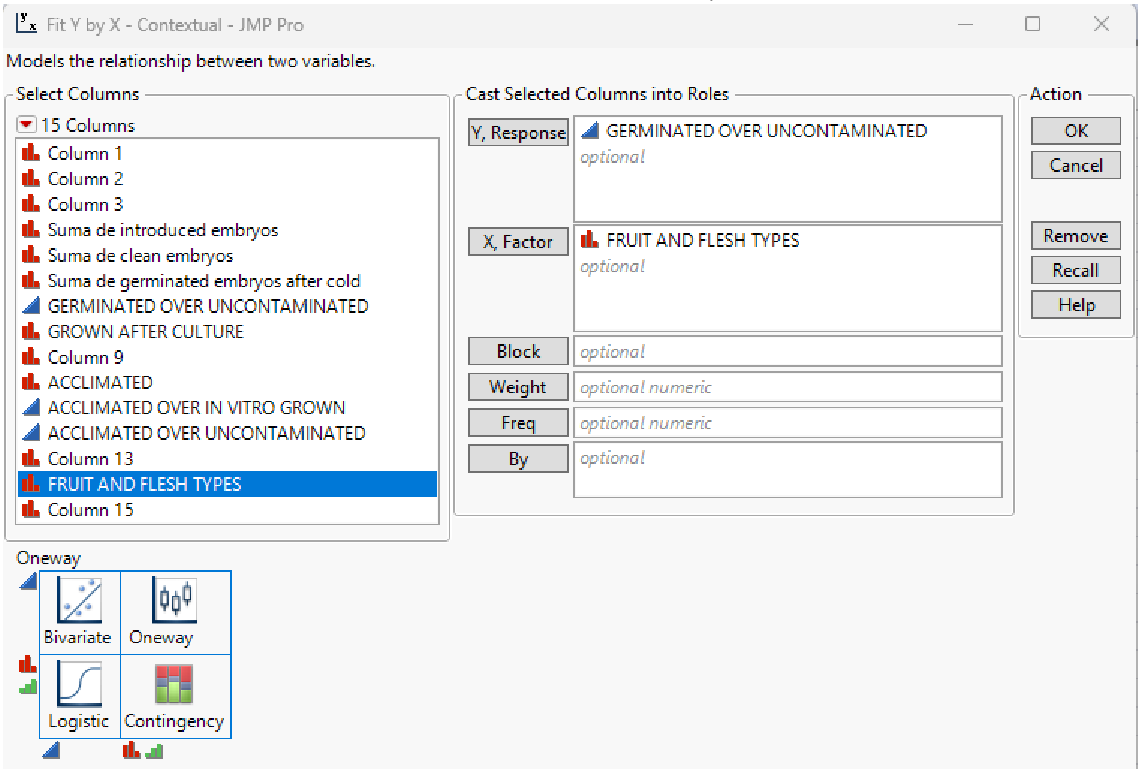

Oneway Analysis of GERMINATED OVER UNCONTAMINATED By FRUIT AND FLESH TYPES

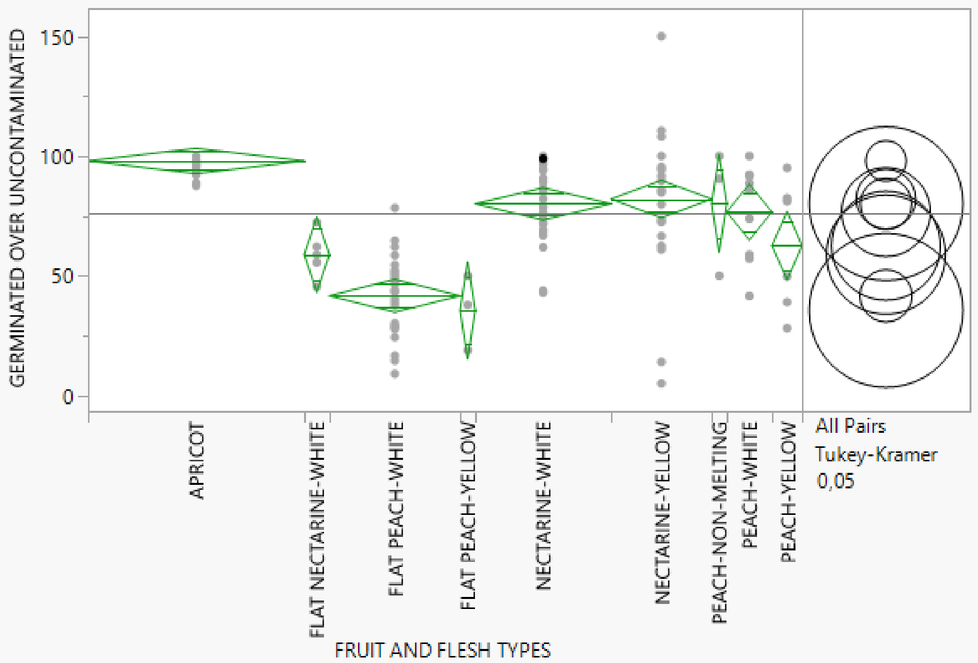

Oneway Anova

Summary of Fit

Analysis of Variance

Source	DF	Sum of Squares	Mean Square	F Ratio	Prob > F
FRUIT AND FLESH TYPES	8	60073,64	7509,20	24,0792	<,0001*
Error	133	41476,61	311,85		
C. Total	141	101550,25			

There are significant differences between Fruit and Flesh Types on Germinated over Uncontaminated Embryos (p<0.0001).

Means for Oneway Anova

Level	Number	Mean	Std Error	Lower 95%	Upper 95%
APRICOT	43	97,9467	2,693	92,620	103,27
FLAT NECTARINE-WHITE	5	58,8504	7,898	43,229	74,47
FLAT PEACH-WHITE	26	41,6457	3,463	34,795	48,50
FLAT PEACH-YELLOW	3	35,5856	10,196	15,419	55,75
NECTARINE-WHITE	27	80,0390	3,399	73,317	86,76
NECTARINE-YELLOW	20	82,0441	3,949	74,234	89,85
PEACH-NON-MELTING	3	80,1587	10,196	59,992	100,33
PEACH-WHITE	9	76,6170	5,886	64,974	88,26
PEACH-YELLOW	6	62,5792	7,209	48,319	76,84

Std Error uses a pooled estimate of error variance

Means Comparisons

Comparisons for all pairs using Tukey-Kramer HSD

Confidence Quantile

q*	Alpha
3,15354	0,05

HSD Threshold Matrix

Connecting Letters Report

Level					Mean
APRICOT	A				97,946699
NECTARINE-YELLOW		B			82,044075
PEACH-NON-MELTING	A	B	C		80,158730
NECTARINE-WHITE		B			80,039005
PEACH-WHITE		B			76,617032
PEACH-YELLOW		B	C	D	62,579161
FLAT NECTARINE-WHITE		B	C	D	58,850428
FLAT PEACH-WHITE				D	41,645746
FLAT PEACH-YELLOW			C	D	35,585586

Levels not connected by same letter are significantly different.

Separation of Germinated over Uncontaminated Embryos means according to Tuckey-Kramer HSD (p=0.05) between Fruit and Flesh Types.

FIGURE 5B

Analyze Fit Y by X

Factors: Fruit and Flesh Types.
Variable: Acclimated Plants over In Vitro Grown Plantlets.

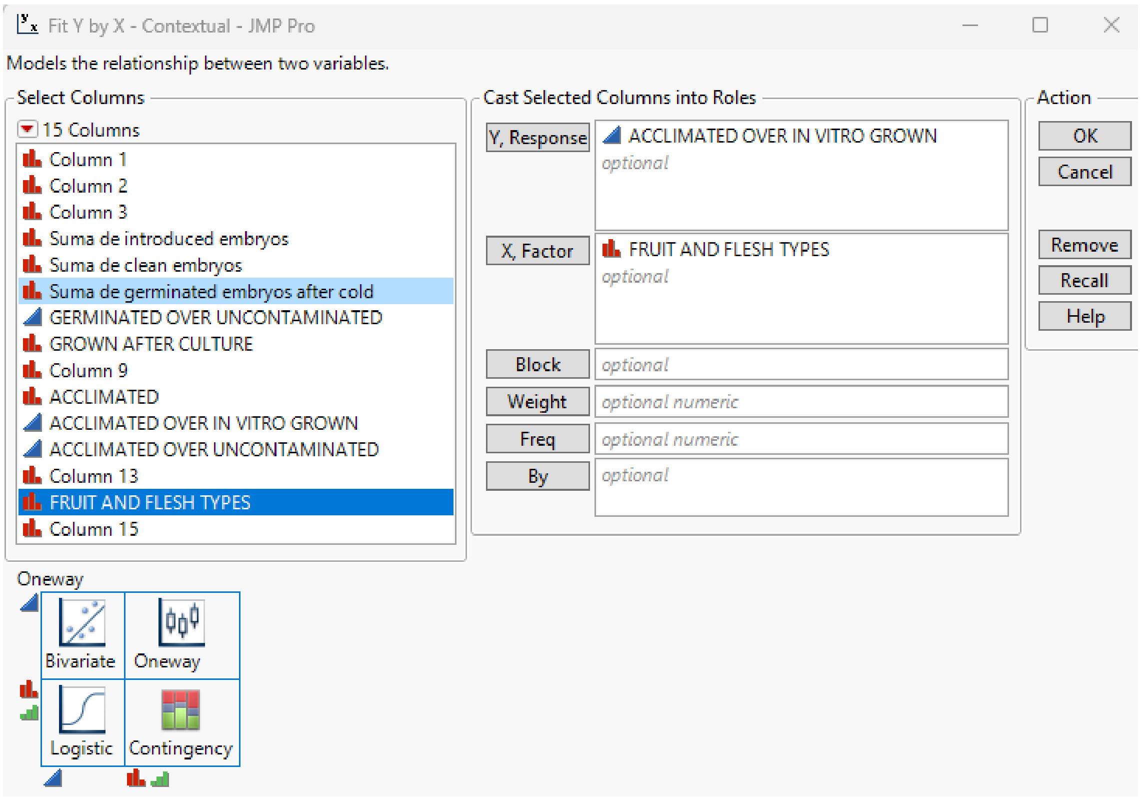

Oneway Analysis of ACCLIMATED OVER IN VITRO GROWN By FRUIT AND FLESH TYPES

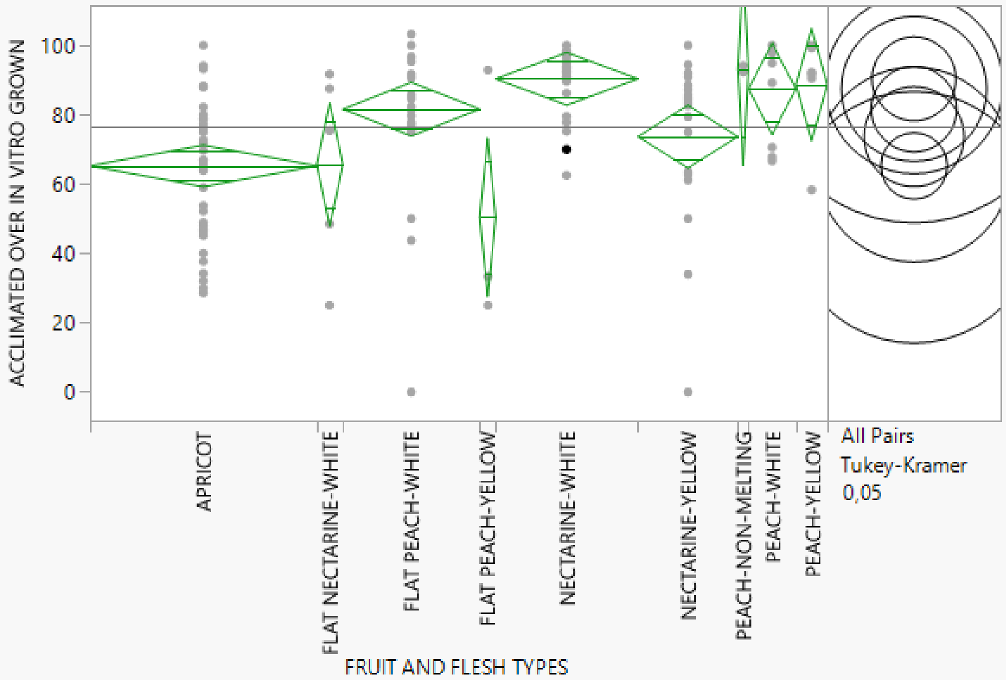

Oneway Anova

Summary of Fit

Analysis of Variance

Source	DF	Sum of Squares	Mean Square	F Ratio	Prob > F
FRUIT AND FLESH TYPES	8	16623,026	2077,88	5,2352	<,0001*
Error	131	51994,112	396,90		
C. Total	139	68617,138			

There are significant differences among Fruit and Flesh Types ($p<0.0001$) on Acclimated Plants over In Vitro Grown Plantlets

Means for Oneway Anova

Level	Number	Mean	Std Error	Lower 95%	Upper 95%
APRICOT	43	65,2112	3,038	59,201	71,22
FLAT NECTARINE-WHITE	5	65,6180	8,910	47,993	83,24
FLAT PEACH-WHITE	26	81,6036	3,907	73,874	89,33
FLAT PEACH-YELLOW	3	50,3968	11,502	27,643	73,15
NECTARINE-WHITE	27	90,3349	3,834	82,750	97,92
NECTARINE-YELLOW	19	73,6670	4,571	64,625	82,71
PEACH-NON-MELTING	2	93,2127	14,087	65,345	121,08
PEACH-WHITE	9	87,4375	6,641	74,300	100,57
PEACH-YELLOW	6	88,6450	8,133	72,555	104,73

Std Error uses a pooled estimate of error variance

Means Comparisons

Comparisons for all pairs using Tukey-Kramer HSD

Confidence Quantile

q*	Alpha
3,15434	0,05

HSD Threshold Matrix

Connecting Letters Report

Level				Mean
PEACH-NON-MELTING	A	B	C	93,212670
NECTARINE-WHITE	A			90,334882
PEACH-YELLOW	A	B	C	88,645007
PEACH-WHITE	A	B	C	87,437452
FLAT PEACH-WHITE	A		C	81,603594
NECTARINE-YELLOW	A	B	C	73,667000
FLAT NECTARINE-WHITE	A	B	C	65,618022
APRICOT		B		65,211210
FLAT PEACH-YELLOW		B	C	50,396825

Levels not connected by same letter are significantly different.

Separation of Acclimated Plants over In Vitro Grown Plantlets means according to Tuckey-Kramer HSD (p=0.05) between Fruit and Flesh Types.

FIGURE 5C

Analyze Fit Y by X

Factors: Fruit and Flesh Types.
Variable: Acclimated Plants over Uncontaminated Embryos.

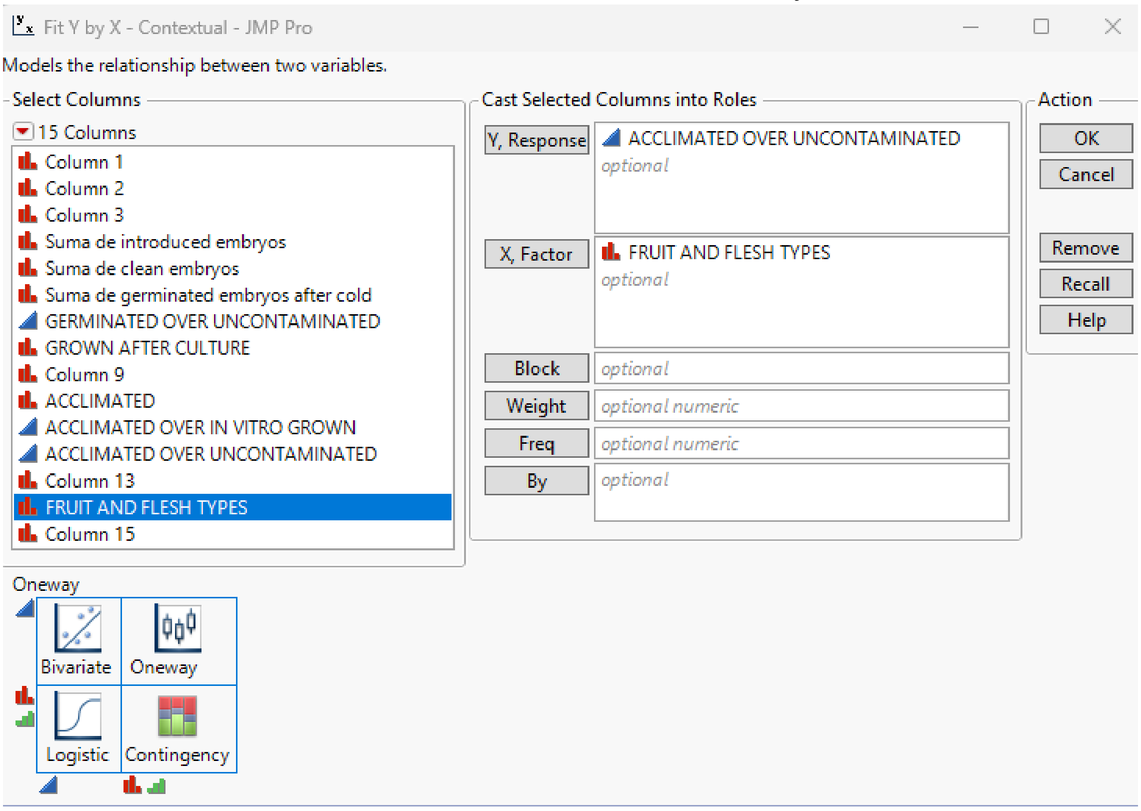

Oneway Analysis of ACCLIMATED OVER UNCONTAMINATED By FRUIT AND FLESH TYPES

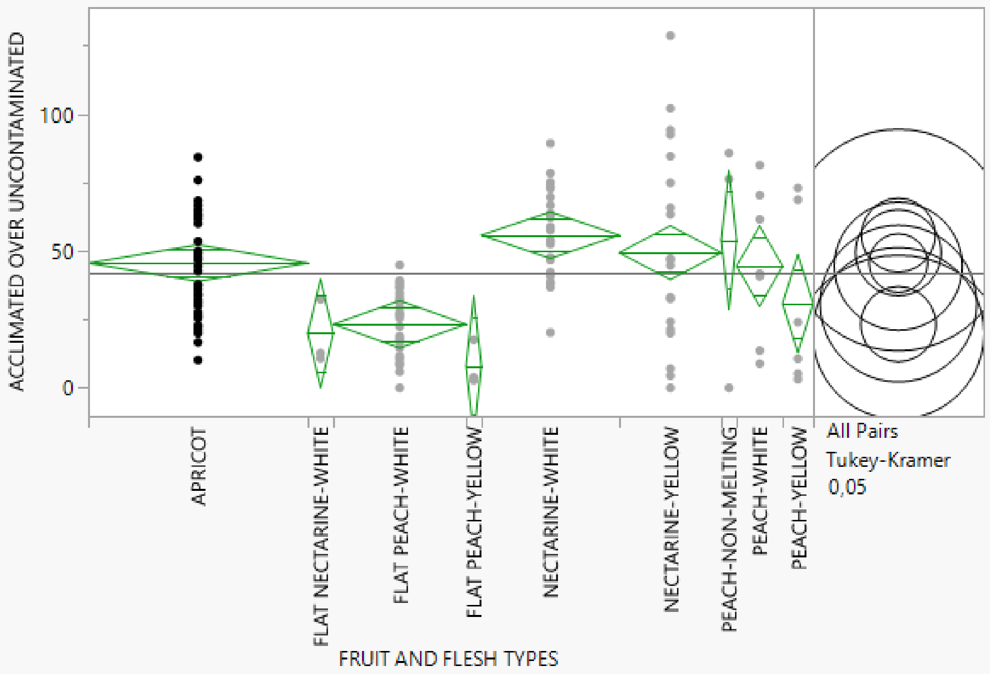

Oneway Anova

Summary of Fit

Analysis of Variance

Source	DF	Sum of Squares	Mean Square	F Ratio	Prob > F
FRUIT AND FLESH TYPES	8	23003,671	2875,46	5,8236	<,0001*
Error	133	65670,220	493,76		
C. Total	141	88673,892			

There are significant differences among Fruit and Flesh Types ($p<0.0001$) on Acclimated Plants over Uncontaminated Embryos.

Means for Oneway Anova

Level	Number	Mean	Std Error	Lower 95%	Upper 95%
APRICOT	43	45,5634	3,389	38,86	52,266
FLAT NECTARINE-WHITE	5	19,9156	9,937	0,26	39,571
FLAT PEACH-WHITE	26	23,2551	4,358	14,64	31,875
FLAT PEACH-YELLOW	3	8,0388	12,829	-17,34	33,414
NECTARINE-WHITE	27	55,7644	4,276	47,31	64,223
NECTARINE-YELLOW	20	49,3439	4,969	39,52	59,172
PEACH-NON-MELTING	3	53,9683	12,829	28,59	79,344
PEACH-WHITE	9	44,4423	7,407	29,79	59,093
PEACH-YELLOW	6	30,7770	9,072	12,83	48,720

Std Error uses a pooled estimate of error variance

Means Comparisons

Comparisons for all pairs using Tukey-Kramer HSD

Confidence Quantile

q*	Alpha
3,15354	0,05

HSD Threshold Matrix

Connecting Letters Report

Level				Mean
NECTARINE-WHITE	A			55,764404
PEACH-NON-MELTING	A	B	C	53,968254
NECTARINE-YELLOW	A		C	49,343945
APRICOT	A		C	45,563401
PEACH-WHITE	A	B	C	44,442252
PEACH-YELLOW	A	B	C	30,777009
FLAT PEACH-WHITE		B		23,255136
FLAT NECTARINE-WHITE		B	C	19,915579
FLAT PEACH-YELLOW		B	C	8,038808

Levels not connected by same letter are significantly different.

Separation of Acclimated Plants over Uncontaminated Embryos means according to Tuckey-Kramer HSD (p=0.05) between Fruit and Flesh Types.
